# Study on Multi-Mode Switching Control Strategy of Active Suspension Based on Road Estimation

**DOI:** 10.3390/s23063310

**Published:** 2023-03-21

**Authors:** Jianze Liu, Jiang Liu, Yang Li, Guangzheng Wang, Fazhan Yang

**Affiliations:** Institute of Mechanical and Automotive Engineering, Qingdao University of Technology, Qingdao 266520, China

**Keywords:** vehicle engineering, road estimation, mode switching, PSO_LQG control, vibration test

## Abstract

In this paper, the least squares method is used to determine the vertical height of the road space domain. Based on the road estimation method, the active suspension control mode switching model is constructed, and the dynamic characteristics of the vehicle in comfort, safety, and integrated modes are analyzed. The vibration signal is collected by the sensor, and the parameters such as vehicle driving conditions are solved for in reverse. A control strategy for multiple mode switching under different road surfaces and speeds is constructed. At the same time, the particle swarm optimization algorithm (PSO) is used to optimize the weight coefficients of LQR control under different modes, and the dynamic performance of vehicle driving is comprehensively analyzed. The test and simulation results show that the road estimation results under different speeds in the same road section are very close to the results obtained by the detection ruler method, and the overall error is less than 2%. Compared with the active suspension controlled by passive and traditional LQR, the multi-mode switching strategy can achieve a better balance between driving comfort and handling safety and stability, and also improve the driving experience more intelligently and comprehensively.

## 1. Introduction

The suspension has an important effect on vehicle driving safety and riding comfort. The traditional active suspension has the restriction that the control characteristics must be the same if the configuration is the same. In the face of complex and changeable road conditions, how to continuously optimize the control algorithm and strategy is a perennial subject of research [1,2]. The vibration signal is collected by the sensor in the process of vehicle driving, and the signal is solved in reverse to perceive the road conditions of vehicle driving. Combined with vehicle driving parameters, the current control mode of the suspension is identified and switched on, and the intelligent control and regulation of the active suspension system are realized with comprehensive consideration of driving conditions [3,4,5].

At present, professional equipment is commonly used in the field of road management to collect international roughness index (IRI) data [6] for quality assessment and monitoring of road management. The traditional manual detection method requires manual measurement tools, which have low efficiency and are greatly affected by human factors, so cannot meet the requirements of traffic control. Special equipment, such as laser sensors or high-speed profilers, also needs to be installed on trucks or trailers [7] rather than conventional passenger vehicles. Although the new compact road profiler [8] can easily collect profile data, evaluate urban public roads, and monitor road roughness conditions in real time, its cost and size are not suitable for use in conventional vehicles. The indirect measurement method, using vehicle dynamics parameters to identify road parameters [9,10], has become a research hotspot in recent years due to advantages such as no special equipment, low cost, easy implementation, and not being affected by rain, snow, and other bad weather. This indirect measurement method collects vehicle dynamics system parameters such as acceleration, displacement or noise of body and chassis, etc. as input variables, and uses neural networks [11,12,13], fuzzy rules [3,14,15], genetic algorithm [16], deep learning [17,18,19], etc., to establish nonlinear mapping models of the parameters to be estimated. Alternatively, the least squares method [20,21,22], Bayesian estimation [23], a Kalman filter [21,24,25,26], or other estimation criteria can be used to identify parameters, estimating the body mass, body side angle, road slope, slip rate, adhesion coefficient, driving style, and road roughness.

The method of establishing a mapping model based on neural networks or fuzzy rules is more commonly used. Liu et al. [11] built a seven-degrees-of-freedom model of a vehicle vibration system and identified the road roughness of B-grade and C-grade roads at the same speed by using the nonlinear autoregressive and exogenous input (NARX) neural network recognition method. The simulation results show that it has ideal recognition accuracy and strong anti-noise ability. In [12], the vehicle inertial response was detected, and then the road profile was reconstructed under an artificial neural network after wavelet decomposition. The results of sensitivity analysis and statistical regression were used to normalize the simulated vehicle response to the driving speed. The roughness of the road profile was obtained by decomposing the combined components of vehicle response by wavelet. However, the architecture of the NARX network for road contour reconstruction requires a lot of data analysis and optimization. Gong et al. [15] proposed establishing T-S fuzzy control rules to realize road grade recognition based on the relative road roughness caused by different road excitation levels. Although the above recognition technology is based on a neural network or fuzzy rules to achieve the estimation of road roughness, solve the road level, and measure time and human factors, it does not consider the cost of special equipment, large volume, or other problems. However, the network architecture used for road contour reconstruction requires a large amount of data for training to establish mapping relationships and rules, and online real-time control is difficult. In addition, acoustic sensors installed on vehicles are used to collect noise during vehicle running [13,19], and methods such as deep learning technology and support vector machine are used to classify road roughness, which can also realize road identification with high accuracy. However, the excessive number of road classifications requires too much algorithmic power to support. Meanwhile, microphones will be affected by wind, water, and dirt, and the location of microphones will also affect the final measurement results.

However, the inverse estimation method based on system dynamic parameters has the advantages of the stable acquisition of dynamic parameters and mapping relationship construction without a large amount of training data, so it has become a research hotspot in recent years. Many scholars apply the Kalman filtering algorithm. For example, Fauriat et al. [25], based on a Renault model, collected parameters such as body speed, acceleration, chassis acceleration, and suspension travel through sensors, and estimated the road profile using Kalman filter theory. Kang et al. [26] used discrete Kalman filtering to estimate road roughness by using the measured values of wheel travel (suspension deflection) and acceleration of the sprung and unsprung mass. The results show that the estimated results are similar to the measured results of the laser profilometer, which proves the correctness of this method. In addition, scholars have also used smartphones as measuring devices to study road estimation. For example, Nagayama et al. [27,28,29] used smartphones that record triaxial acceleration, angular velocity, and GPS signals as measuring devices. The combined enhanced Kalman filter, Robbins–Monro algorithm, and Rauch–Tung–Striebel method were used to estimate the road profile. Although the above method has a better inverse solution to the road parameters, it has a strong dependence on the hardware computing force and does not consider the influence of different road and speed coupling. Heyns et al. [23] noticed the impact of vehicle speed on road surface estimation, collected rear wheel acceleration and drive shaft-wheel speed data through the on-board data collection system, established regression functions based on the observation results of the road section based on Bayesian parameter estimation method, realized the estimation of road surface roughness, and considered the impact of different vehicle speeds on the accuracy of estimation. This method avoids the influence of misjudgment caused by driving speed on road profile estimation, but performs a probability estimation by establishing data intervals and does not realize the true decoupling of vehicle speed.

At present, most of the optimization schemes of active and semi-active suspension control strategies are weight coefficient optimization for a single working condition. In the actual driving process of vehicles, road conditions are complex and changeable, and the change in vehicle conditions will affect the actual control effect [30,31]. Road working condition parameters can be obtained to optimize active suspension control and obtain better control effects under different road conditions and speeds, which can better improve vehicle comfort and ride comfort [5,32]. Therefore, it is necessary to establish a mode-switching control strategy for different road conditions.

For different mode-switching control in vehicle running, many scholars have performed related research on ride comfort. For example, Zhang et al. [33], Peng et al. [34], and Tao et al. [35] studied the comfort of seat suspension and vehicle suspension. The authors of [32] constructed a multi-mode active seat suspension, which controlled three mode switches according to vertical seat acceleration and suspension dynamic driving signals. Simulation results showed that the ride comfort was improved compared with passive seat suspension. Peng et al. [34] designed a multi-mode switching controller based on a composite suspension with parallel electromagnetic and magnetorheological shock absorbers to improve ride comfort. The simulation and test results showed that the multi-mode switching controller can effectively switch between the working modes of parallel composite electromagnetic suspension and improve adaptability to different road conditions. Tao et al. [35] proposed a novel semi-active suspension with multiple damping modes, which realized multi-mode switching damping characteristics by changing the discrete state of two high-speed on-off solenoid valves. The simulation results show that the proposed method has better vibration-damping performance than passive suspension and traditional PID semi-active suspension. However, the above multi-mode switching control of active suspension is mainly aimed at riding comfort, without considering other vehicle dynamics evaluation indexes.

To verify the superiority of multi-mode switching control strategy for different driving conditions under the automobile dynamics evaluation index, the research team of Wang et al. [36,37] conducted a large number of studies. They used the active suspension equipped with a linear motor to propose a mode-switching method to coordinate the relationship between energy recovery and suspension vibration. In simulations and testing, the root means square of body vertical acceleration and tire dynamic load decreased by 21.35% and 4.24%, respectively, under the active control mode at the speed of 20 m/s on the C-grade road surface, which is far better than the passive suspension. However, this study only discusses the comparison between traditional active suspension and passive suspension under multi-mode switching on a single road surface and does not discuss the optimization control of active suspension in different modes under different roads. Subsequently, in the latest study of Wang et al. [38], they studied a semi-active suspension switching control strategy based on road profile estimation. Firstly, the mapping relationship between different driving conditions and vehicle dynamic performance requirements was constructed, and then the sky-hook control was optimized by the cuckoo search algorithm to achieve control optimization and automatic switching under different road conditions. The bench test results showed that the ride comfort and handling stability improved, which verified the superiority of the handover control strategy under the road estimation. However, the road estimation method based on the mapping relationship established by a large number of training sets cannot inversely solve the actual road excitation and then decouple the speed to get the actual road power spectral density value. Meanwhile, the semi-active suspension was slightly less effective at control than the active suspension.

Therefore, we constructed a multi-mode switching control strategy based on road estimation. Firstly, the vehicle dynamic parameters and driving speed were collected, the road excitation was estimated by the improved least square method, and the speed was deconstructed to obtain the power spectral density (PSD) value. This method does not require a large amount of data for training—the least squares method based on the calculation force requirements requires few data—and the monitoring of sudden changes in the road surface is good. At the same time, the unevenness parameter, which is not affected by the speed, is obtained by decoupling the speed variable. Based on the estimation of the corresponding road conditions, a variety of switching modes was constructed, and the driving modes of the corresponding conditions were determined by establishing different threshold decision ranges. To “suit the medicine to the illness”, the particle swarm optimization algorithm (PSO) was used to re-optimize the weight coefficient in LQR control according to different working conditions, to realize the intelligent adjustment of the control parameters of the active suspension system, which is of great significance to comprehensively improve the driving experience of vehicles. The structure of this paper is as follows: In Section 2, the mathematical model of road excitation based on the uniform change of speed and road is constructed, and the correctness of the mathematical model is verified by simulating the excitation change of a sudden change of road under four kinds of road and three speeds (low, medium, or high). In Section 3, the estimation model of road power spectral density based on the improved least square method is proposed, and the related problems of acquisition signal processing are specified. In Section 4, four switching modes are discussed after road surface and vehicle parameters are obtained, and four switching strategies are constructed according to the judgment of different threshold ranges. In Section 5, the mathematical model of the particle swarm optimization algorithm is constructed and applied to the optimization solution of the LQR control coefficient under different working conditions. In Section 7, the feasibility and accuracy of the multi-mode switching control strategy based on road estimation are verified by experiments. These are discussed and conclusions are drawn in Section 7.

## 2. Dynamic Model Analysis of Suspension System under a Variable Road Surface

### 2.1. Establishment of 1/4 Vehicle Model with Two Degrees of Freedom

**Figure 1:**
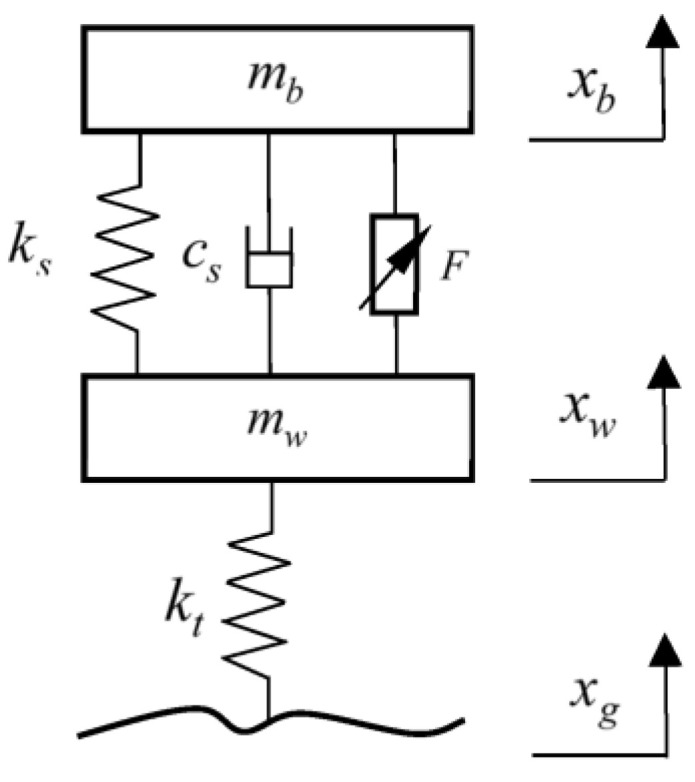
Two-degrees-of-freedom model of 1/4 vehicle.

To simplify the model, a two-degrees-of-freedom model of a 1/4 vehicle with an energy-regenerative active suspension system was established, as shown in Figure 1 [39,40]. The energy-regenerative suspension system consists of an actuator conversion mechanism, a transmission mechanism, and a motor.

According to the automobile dynamics theory, the dynamic differential equation of a 1/4 vehicle model with two degrees of freedom was established as follows:

(1) $$\{\begin{array}{c} m_{b}{\ddot{x}}_{b}=-k_{s}\left(x_{b}-x_{w}\right)-c_{s}\left({\dot{x}}_{b}-{\dot{x}}_{w}\right)+F\\ m_{w}{\ddot{x}}_{w}=k_{s}\left(x_{b}-x_{w}\right)+c_{s}\left({\dot{x}}_{b}-{\dot{x}}_{w}\right)-k_{t}\left(x_{w}-x_{g}\right)-F \end{array},$$

where *m_b_* is the body mass; *x_b_* is body displacement; *m_w_* is the wheel mass; *x_w_* is the wheel displacement; *x_g_* is the road input; *k_s_* is the suspension stiffness; *c_s_* is the suspension damping; *k_t_* is the tire stiffness; and *F* is for active control.

### 2.2. Establishment of Variable Road Suspension State Equation Model

According to the statistical characteristics, road roughness is usually divided into eight grades. According to the random data of road roughness measured, the value range and mean value of the vertical displacement power spectral density *G_q_*(*n*) of different levels of the road surface are obtained by the algorithm. According to the standards of ISO/TC 108/SC2N67 and GB7031 “Vehicle Vibration Input—Road Flatness Representation”, the value range and mean value of vertical displacement power spectral density *G_q_*(*n*_0_) of the road at different levels are listed, as shown in Table 1 [4].

In traditional research, the study of random road suspension systems usually selects a B-grade or C-grade road surface for simulation and testing under the fixed parameters of a speed of 10 m/s or 20 m/s. However, the actual driving condition of the car is relatively complex. When the vehicle suddenly moves from one level of the road surface to another level, the spring stiffness coefficient k and damping c of the traditional suspension will not change. Even for semi-active and active suspensions, most only have a fixed output force or damping change to improve ride comfort and comfort. Some researchers have proposed improving vehicle driving conditions by changing the fixed value damping of multiple groups of damping variables. In this paper, driving conditions are improved by intelligent identification of road driving conditions and reasonable distribution of active suspension control force. Especially when the level of the road suddenly changes, the signal collected by the sensor can reasonably analyze the information of the change in the road level. Based on this, active suspension allows for better control to achieve improvement in vehicle ride comfort.

However, according to the different road conditions and speeds, the real-time body acceleration, suspension dynamic travel, and tire dynamic displacement will have a large gap. Improving the accuracy of road identification may solve the problem that the active suspension still cannot actively search for optimization control because the road surface has relatively large fluctuation due to the failure to reach the threshold switching point because there is only one threshold. For example, when driving at low speed or high speed on a high-grade road surface, the driving experience is very different. However, considering the division of different road grades, and real-time changes in speed, this creates a huge amount of data for computation, and poses great difficulty in the selection of mode switching threshold. Therefore, to reduce and simplify the number of thresholds and the complexity of classification, four grades were set, A, B, C, and D, the D−H grades is considered to be the switching threshold of the D−grade. This mainly takes into account the fact that common urban roads involving D-grade and above are relatively rare. That is, it improves the precision of the classification to a certain extent, and avoids the miscellaneous operations and responses brought about by excessive fine division to the whole system.

Road roughness is used as the vibration input during vehicle running and is usually represented by the vertical displacement power spectral density of the road surface *G_q_*(*n*). It decreases with an increase in spatial frequency *n* or a decrease in wavelength *λ*, which describes the statistical characteristics of pavement spatial displacement, while the time history of pavement roughness is regarded as a stationary random process. Therefore, according to the ISO/TC108 standard, *G_q_*(*n*) is expressed by a power function:

(2) $$G_{q}\left(n\right)=G_{q}\left(n_{0}\right){\left(n/n_{0}\right)}^{-W},$$

where *G_q_*(*n*_0_) is the road roughness coefficient (m^3^); *W* is the frequency index; *n* is the spatial frequency (m^−1^); and *n_*0*_* is the reference space frequency (m^−1^). 

In the actual process of vehicle movement, speed *v_*0*_* is also a factor of vehicle vibration input. The power spectral density *G_q_*(*n*_0_) at the reference spatial frequency *n_*0*_* is converted into the time-frequency spectral density *G_q_*(*f*); where *f* = *v_*0*_n*, when the frequency index *W* = 2, Equation (3) gives the time-frequency spectral density *G_q_*(*f*):

(3) $$G_{q}\left(f\right)=G_{q}\left(n\right)/v_{0}=G_{q}\left(n_{0}\right)n_{0}^{2}v_{0}/f^{2}.$$

The random road profile adopted in this paper is generated by unit white noise through the filter. The expression of filtered white noise is as follows:

(4) $${\dot{x}}_{g}\left(t\right)=-2\pi f_{0}x_{g}\left(t\right)+2\pi n_{0}\sqrt{G_{q}\left(n_{0}\right)v_{0}}w\left(t\right),$$

where *w*(*t*) is the time-domain signal of unit white noise; *f*_0_ is the lower cutoff time-frequency; and *x_g_*(*t*) is the time-domain signal of the road spectrum.

When using the space state equation of a 1/4 suspension system to analyze the evaluation index of vehicle suspension performance, the fixed parameters are limited by the matrix form of the equation. It is difficult to change the fixed parameter to a variable parameter for the system motion equation and road input equation. In other words, in the adopted road vertical displacement power spectral density, the road roughness coefficient *G_q_*(*n*_0_) is a certain value parameter at the input, while the establishment of a variable road surface model requires *G_q_*(*n*_0_) to change with time at the input and be a variable.

Through observation, we found that in filtering white noise via Equation (4), each parameter is a single continuous fixed value that does not change over time. According to this principle, we changed the single continuous fixed value, changing with time *t*, into a continuous variable value, thus realizing the change from a fixed parameter to a variable parameter in the space state equation of the system:

(5) $$\begin{array}{l} G_{qi}\left(n_{0}\right)=\left[\begin{array}{cc} G_{q1}\left(n_{0}\right) & t_{1}\\ G_{q2}\left(n_{0}\right) & t_{2}\\ G_{q3}\left(n_{0}\right) & t_{3}\\ \vdots & \vdots \\ G_{qn}\left(n_{0}\right) & t_{n} \end{array}\right];v_{j}=\left[\begin{array}{cc} v_{1} & t_{1}\\ v_{2} & t_{2}\\ v_{3} & t_{3}\\ \vdots & \vdots \\ v_{n} & t_{n} \end{array}\right];\\ {\dot{X}}_{g}\left(t\right)=-2\pi f_{0}X_{g}\left(t\right)+2\pi n_{0}\sqrt{G_{qi}\left(n_{0}\right)•v_{j}}•W\left(t\right) \end{array}$$

where i,j=1,2,3…n.

According to the vehicle system dynamics, the system motion equation and road surface input equation were written into a matrix form by combining Equations (1), (3), and (5)—that is, the system space state equation can be written as follows:

(6) $$\dot{X}=AX+BU+HW,$$

where $X={\left[{\dot{x}}_{b},{\dot{x}}_{w},x_{b},x_{w},x_{g}\right]}^{T}$ is the system state vector, U=[F(t)] is the control input matrix of active suspension, and W=[w(t)] is the Gaussian white noise input matrix.

By writing the system motion equation in matrix form, we can obtain the following:

$$A=\left[\begin{array}{ccccc} -c_{s}/m_{b} & c_{s}/m_{b} & -K_{s}/m_{b} & K_{s}/m_{b} & 0\\ c_{s}/m_{w} & -c_{s}/m_{w} & K_{s}/m_{w} & \left(-K_{t}-K_{s}\right)/m_{w} & K_{t}/m_{w}\\ 1 & 0 & 0 & 0 & 0\\ 0 & 1 & 0 & 0 & 0\\ 0 & 0 & 0 & 0 & -2\pi f_{0} \end{array}\right];B=\left[\begin{array}{c} 1/m_{b}\\ -1/m_{w}\\ 0\\ 0\\ 0 \end{array}\right];H=\left[\begin{array}{c} 0\\ 0\\ 0\\ 0\\ 2\pi n_{0}\sqrt{G_{q}\left(n_{0}\right)v_{0}} \end{array}\right]$$

Similarly, the output equation of the system is:

(7) Y=CX+DU.

In the design of vehicle suspension, body acceleration (BA), suspension working space (SWS), and dynamic wheel displacement (DTD) (or dynamic tire load (DTL)) are usually taken as the important performance evaluation indexes. It should be noted that DTL and DTD are two indicators that describe the grip performance of tires, evaluating force disturbance and position disturbance, respectively. The two are linearly related, and the scale factor is the tire vertical stiffness, which is DTL = *K_s_* · DTD. The magnitude of the statistical eigenvalue of DTD is different for different road surfaces. The random excitation from the pavement displacement can be considered as a perturbation to the tire droop force input according to Hooke’s law and is reflected in the signal characteristic steady-state feature as the pavement grade changes [41,42]. In this paper, DTD is used as the performance index because it is easier to obtain the observed data. So that is the choice $Y={\left[{\ddot{x}}_{b},x_{b}-x_{w},x_{w}-x_{g}\right]}^{T}$. Similarly, Equation (7) is written in matrix form:

$$C=\left[\begin{array}{ccccc} -c_{s}/m_{b} & c_{s}/m_{b} & -K_{s}/m_{b} & K_{s}/m_{b} & 0\\ 0 & 0 & 1 & -1 & 0\\ 0 & 0 & 0 & 1 & -1 \end{array}\right];D=\left[\begin{array}{c} 1/m_{b}\\ 0\\ 0 \end{array}\right].$$

### 2.3. Example Analysis of Changing Road

The road surface displacement *x_g_*(*t*) is transformed once every 20 s, and the road surface displacement comparison diagram of A to D is generated according to the roughness coefficient *G_q_*(*n*_0_) of each grade of the road surface in turn in Table 1 [43]. Where vehicle speed *v*_0_ = 20 m/s, *w*(*t*) is the time-domain signal of unit white noise, and the specific vehicle parameters are as shown in Table 2.

**Figure 2:**
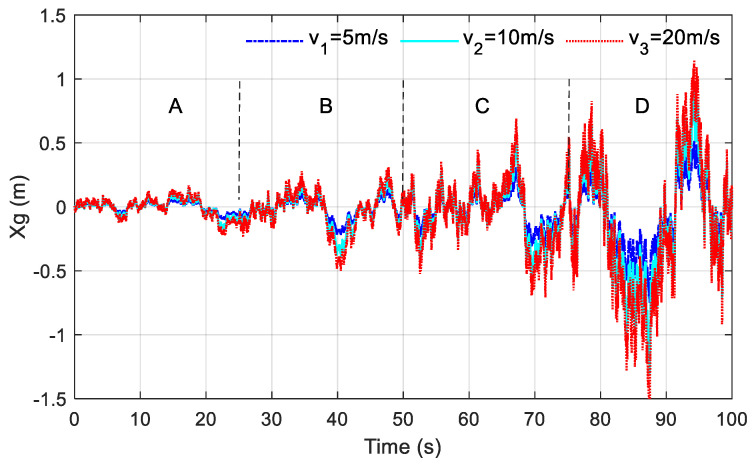
Comparison of road displacement changes at different speeds of A–D grade road.

As shown in Figure 2, the road excitation, changing every 25 s from A-grade to D-grade, is compared, where the blue solid line, blue-green dotted line, and red dotted line are the changes at 5 m/s, 10 m/s, and 20 m/s, respectively. It can be seen that, no matter what the speed on the A-grade road surface, the advantage brought by the road roughness to the vehicle is relatively small, and the reciprocating vibration is ±0.2 m. The vibration amplitude increases step by step from A-grade to D-grade, and it can be seen that road roughness is one of the main factors affecting the road excitation input. At the same time, it is also noted that with the same road surface, different speed also affects the road surface excitation input to a certain extent. The instantaneous vibration displacement at different speeds on each level of the road surface alternates positively and negatively, seemingly in a disordered manner, but with certain statistical rules, basically conforming to the law of random vibration.

**Figure 3:**
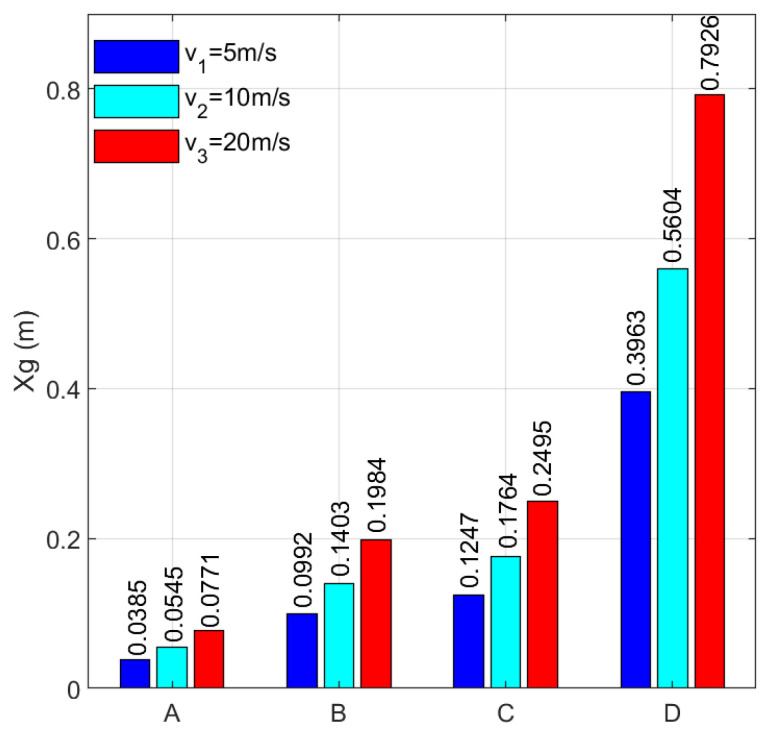
Comparison of RMS values of road displacement under different working conditions.

For quantitative analysis, root-mean-square value processing was carried out on the displacement of each road level at each speed to obtain the comparison data, as shown in Figure 3, where the blue, blue-green, and red bar graphs represent the road excitation RMS values at 5 m/s, 10 m/s, and 20 m/s, respectively. The law of its numerical variation is consistent with that shown in Figure 2. Through quantitative analysis, it can be found that the road excitation RMS value under each road grade has certain rules. When the road surface is equal, the RMS value of the three speeds has a certain proportional relationship— that is, the RMS value at 20 m/s is about twice that at 5 m/s, and the RMS value at 10 m/s is about 1.41 times that at 5 m/s. These two multiples are exactly equal to the values of $\sqrt{v_{3}/v_{1}}$ and $\sqrt{v_{2}/v_{1}}$, which can be easily explained by Equations (4) and (5), confirming the correctness of the solution. It also provides a basis for the division of handover policies in Section 4.

## 3. Variable Road Identification Method

### 3.1. Construction of Road Parameter Acquisition Model

Tire displacement *x_g_* is the fluctuation of vertical displacement at the contact point between the tire and the road surface, which is difficult to collect directly. Therefore, vehicle speed *v*_0_, body acceleration ${\ddot{x}}_{b}$, and chassis acceleration ${\ddot{x}}_{w}$ are taken as input data. By analyzing the data characteristics of the input and output of the suspension system, it can be concluded that the suspension system is discrete, so the least squares estimation criterion can be used to estimate the system parameters. Since 1/4 of the vehicle model identification equations are nonlinear equations, the problem becomes a functional extreme value problem including differential and integral equations, and there is no analytical solution. The iterative approximation of a linear equation is usually used to solve this kind of nonlinear problem.

**Figure 4:**
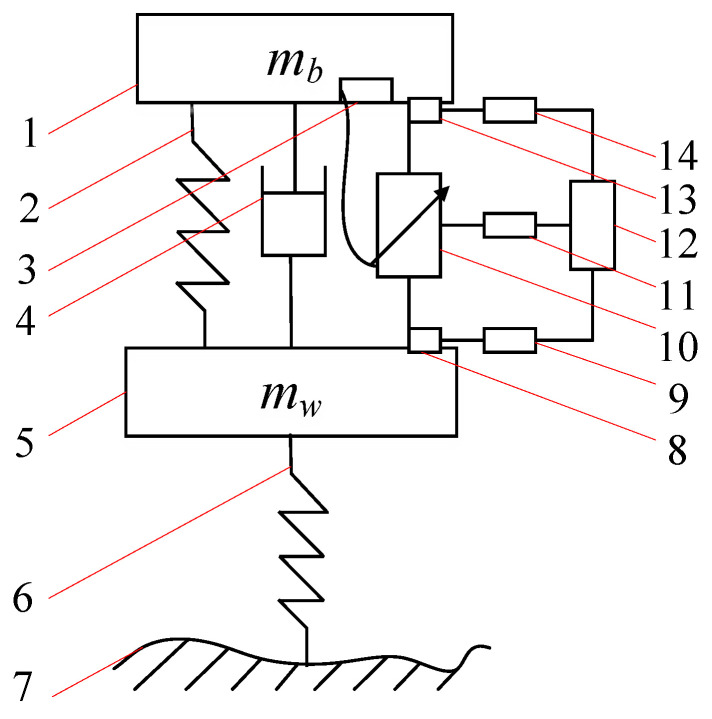
Structural drawing of energy regenerative active suspension. 1. Body 2. Damping spring 3. Storage/energy supply module 4. Suspension damping 5. Wheel 6. Tire equivalent spring 7. Road 8/13. Sensor 9/14. DAC 10. Ball screw type shock absorber 11 ADC 12. ECU.

To realize the data collection of road identification and complete the control strategy of suspension multi-mode switching according to the information on the road surface, the project team built an active suspension structure, as shown in Figure 4. It is composed of a sensor, Analog–Digital Converter (ADC), Digital–Analog Converter (DAC), Electronic Control Units (ECU), damping spring, damping, ball screw shock absorber, energy storage module, etc. [21,40,44].

The vibration data of the body and chassis are collected by the acceleration sensor; then the electric signal is converted into a digital signal by the ADC and transmitted to the Electronic Control Unit (ECU). The ECU analyzes the converted digital signal and identifies the current road state of the vehicle by combining the vehicle’s traveling speed parameter information. ECU then determines the corresponding mode of the current running state by using a multi-mode switching control strategy and optimizes the Linear Quadratic Gaussian (LQG) active suspension controller by Particle Swarm Optimization. The optimized control parameters are transmitted to the ball screw damper through the digital-to-analog converter to realize the optimized control for different road surfaces in various modes.

### 3.2. Construction of Variable Road Identification Model

Firstly, the observation equation of the discrete linear system should be established:

(8) Y=H⋅θ+V,

where $Y\in R^{m\times 1}$ is the observation vector; $\theta \in R^{n\times 1}$ is the parameter to be estimated; measurement noise $V\in R^{m\times 1}$, zero mean white noise; observation matrix $H\in R^{m\times n}$.

If one has *i* observations, and *j* observations, m is equal to *i × j*. According to the linear algebra theory, the number of parameters to be estimated is fewer than the number of observed data, that is, *m ≥ n*. Since the system parameters do not change during operation, the observation matrix can be written as follows:

(9) $$H={\left[\begin{array}{cccc} h\left(1\right) & h\left(2\right) & \cdots & h\left(j\right) \end{array}\right]}^{T}\in R^{m\times n}.$$

The equation of the *p* observed quantity can be written as follows:

(10) $$y_{p}=h_{p1}\cdot \theta_{1}+h_{p2}\cdot \theta_{2}+\cdots +h_{pn}\cdot \theta_{n}+v_{p}.$$

It can be concluded that *h_pk_* in the observation equation is the amplification coefficient of the parameter *θ_k_*, and the larger *h_pk_* is, the greater the contribution of the parameter *θ_k_* to the observed quantity. Therefore, the observation matrix $H\in R^{m\times n}$ can also be used as the parameter sensitivity coefficient matrix.

The least-squares estimation criterion is used to determine the estimated value of the parameter $\hat{\theta }$, which requires the quadratic function of the observation error to be minimum. Then, for the *v_p_* of each observation equation, the minimum quantity is obtained:

(11) $$J=V^{T}\cdot V={\left[H\cdot \theta -Y\right]}^{T}\left[H\cdot \theta -Y\right]=\min.$$

Considering that the function of quadratic form is a scalar, it can be expanded to:

(12) $$J={\hat{\theta }}^{T}\left(H^{T}H\right)\hat{\theta }-2Y^{T}H\hat{\theta }+Y^{T}Y.$$

At the extreme point ∂J/∂θ=0, the parameter estimate under the least squares estimation criterion can be obtained:

(13) $$\hat{\theta }={\left[H^{T}H\right]}^{-1}H^{T}Y.$$

Random road profiles are generated by a unit white noise filter, as shown in Equation (4). The vehicle dynamics theory establishes the differential equation of 1/4 vehicle model dynamics, as shown in Equation (1). Thus, it can be obtained that the output variables of the suspension system are $X={\left[{\dot{x}}_{b},{\dot{x}}_{w},x_{b},x_{w},x_{g}\right]}^{T}$, while *G_q_*(*n*_0_), *v*_0_, *w*(*t*), and *F* are input variables, and *f*_0_, *n*_0_, *m_b_*, *m_w_*, *k_s_*, *k_t_*, and *c_s_* are quantitative parameters.

The input variable *G_q_*(*n*_0_) is directly related to the grade of the road surface, so the specific conditions of the road surface can be known only by the value of this variable. However, it is also noted that *G_q_*(*n*_0_) is the input variable, and the application condition of the least squares estimation criterion requires that the parameters to be estimated should be quantitative.

### 3.3. Establishment of the Identification Model

To solve this problem, the following assumptions were made in this paper: (1) To smooth the data processing, the sampling length *l_r_* was set as the five data points corresponding to the road excitation, following the five-point cubic filtering principle. (2) To reasonably and comprehensively reflect the grade of the whole road, a minimum evaluation length *l_n_* should be specified in the evaluation: *l_n_ = 5l_r_*. (3) To reduce the calculation amount, reduce the error caused by the random road. The RMS value of the observation variable within the assessed length is taken as an observation quantity for calculation.

Within the assessed length, the sampling frequency of the random road profile was 200 Hz. Within a rated length, the sampling time *t_n_* = 0.125 s. Since road excitation is a stationary random process of various states, the RMS value of *G_q_*(*n*_0_) in this sampling length can be regarded as a quantitative parameter to be estimated [45].

Note that Equation (5) is a group of associated formulas with three parameters, so the combination of similar terms can be simplified. First, Equation (1) is combined with similar terms, and the least squares estimation criterion is applied to solve the parameters.

(14) $$\{\begin{array}{c} {\ddot{x}}_{b}=-\frac{m_{w}}{m_{b}}{\ddot{x}}_{w}+\frac{k_{t}}{m_{b}}x_{g}-\frac{k_{t}}{m_{b}}x_{w}\\ {\dot{x}}_{g}+2\pi f_{0}x_{g}=2\pi n_{0}\sqrt{v_{0}}w\left(t\right)\cdot \sqrt{G_{q}\left(n_{0}\right)} \end{array}$$

The observation equation can be obtained:

(15) Y=H⋅θ+V,

where $Y={\left[y_{1},y_{2},\cdots ,y_{m}\right]}^{T}={\left[{\ddot{x}}_{b1},{\ddot{x}}_{b2},\cdots ,{\ddot{x}}_{bm}\right]}^{T}$; $\theta ={\left[1,\theta_{1},\theta_{2}\right]}^{T}={\left[1,x_{g},x_{w}\right]}^{T}$; $H={\left[h_{1},h_{2},\cdots ,h_{m}\right]}^{T}$; $h_{i}=\left[-\frac{m_{w}}{m_{b}}{\ddot{x}}_{wi},\frac{k_{t}}{m_{b}},-\frac{k_{t}}{m_{b}}\right]$. The available observation equation is:

(16) $$\left[\begin{array}{c} y_{1}\\ y_{2}\\ \cdots \\ y_{m} \end{array}\right]=\left[\begin{array}{c} \begin{array}{ccc} -\frac{m_{w}}{m_{b}}{\ddot{x}}_{w1} & \frac{k_{t}}{m_{b}} & -\frac{k_{t}}{m_{b}} \end{array}\\ \begin{array}{ccc} -\frac{m_{w}}{m_{b}}{\ddot{x}}_{w2} & \frac{k_{t}}{m_{b}} & -\frac{2k_{t}}{m_{b}} \end{array}\\ \cdots \\ \begin{array}{ccc} -\frac{m_{w}}{m_{b}}{\ddot{x}}_{wm} & \frac{k_{t}}{m_{b}} & -\frac{mk_{t}}{m_{b}} \end{array} \end{array}\right]\left[\begin{array}{c} 1\\ x_{g}\\ x_{w} \end{array}\right]+\left[\begin{array}{c} v_{1}\\ v_{2}\\ \cdots \\ v_{m} \end{array}\right].$$

Put into Equation (13), the estimated values of parameters under the least squares estimation criterion are:

(17) $$\hat{\theta }=\{\left[\begin{array}{cccc} -\frac{m_{w}}{m_{b}}{\ddot{x}}_{w1} & -\frac{m_{w}}{m_{b}}{\ddot{x}}_{w2} & \ldots & -\frac{m_{w}}{m_{b}}{\ddot{x}}_{wm}\\ \frac{k_{t}}{m_{b}} & \frac{k_{t}}{m_{b}} & \ldots & \frac{k_{t}}{m_{b}}\\ -\frac{k_{t}}{m_{b}} & -\frac{2k_{t}}{m_{b}} & \ldots & -\frac{mk_{t}}{m_{b}} \end{array}\right]{\left[\begin{array}{c} \begin{array}{ccc} -\frac{m_{w}}{m_{b}}{\ddot{x}}_{w1} & \frac{k_{t}}{m_{b}} & -\frac{k_{t}}{m_{b}} \end{array}\\ \begin{array}{ccc} -\frac{m_{w}}{m_{b}}{\ddot{x}}_{w2} & \frac{k_{t}}{m_{b}} & -\frac{2k_{t}}{m_{b}} \end{array}\\ \ldots \\ \begin{array}{ccc} -\frac{m_{w}}{m_{b}}{\ddot{x}}_{wm} & \frac{k_{t}}{m_{b}} & -\frac{mk_{t}}{m_{b}} \end{array} \end{array}\right]\}}^{-1}\left[\begin{array}{cccc} -\frac{m_{w}}{m_{b}}{\ddot{x}}_{w1} & -\frac{m_{w}}{m_{b}}{\ddot{x}}_{w2} & \ldots & -\frac{m_{w}}{m_{b}}{\ddot{x}}_{wm}\\ \frac{k_{t}}{m_{b}} & \frac{k_{t}}{m_{b}} & \ldots & \frac{k_{t}}{m_{b}}\\ -\frac{k_{t}}{m_{b}} & -\frac{2k_{t}}{m_{b}} & \ldots & -\frac{mk_{t}}{m_{b}} \end{array}\right]\left[\begin{array}{c} y_{1}\\ y_{2}\\ \ldots \\ y_{m} \end{array}\right]$$

As can be seen from Equation (14), there is no driving speed effect in the first equation. After solving the road excitation, the estimated value of *G_q_*(*n*_0_) can be obtained only by decoupling the speed of the second equation. According to the grade division of all levels of pavement in Table 1, the multi-mode division and switching of the suspension system based on pavement estimation are discussed.

## 4. Research on Multi-Mode Switching Strategy

### 4.1. Suspension Function Module Construction

**Figure 5:**
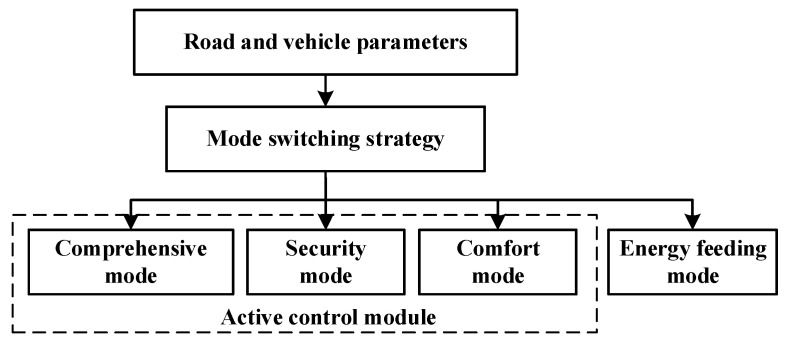
Multi-mode switching control strategy.

The intelligent suspension provides four functional modes as shown in Figure 5. The energy regenerative mode can be used to store the energy generated by the vibration of the vehicle suspension, especially suitable for the range extension of electric vehicles; the active control module is used to improve the vehicle riding comfort and safety, and improve the vehicle performance [33,35,36].

The mode-switching control strategies for different road surfaces are introduced as follows:

(1) Comprehensive mode

The Linear Quadratic Regulator (LQR) algorithm is used to improve the quadratic regulator and ride safety of vehicles and improve the comprehensive performance of vehicles.

(2) Security mode

The LQG Optimization algorithm of Particle Swarm Optimization (PSO) was adopted to optimize the tire dynamic displacement as the key object to improve the tire grip ability and enhance driving safety.

(3) Comfort mode

Using the Particle Swarm Optimization (PSO) LQG optimization algorithm, body acceleration is the focus of the optimization object, while improving the car riding comfort, trying not to sacrifice the tire grip ability, to ensure driving safety.

(4) Energy regenerative mode

The motor of the active suspension stops the main power output and converts the energy generated by the vibration of the suspension into electric energy, which is stored in the energy storage device. It can be switched back on when driving on a good road surface, so is suitable for continuous driving of vehicles. Energy regenerative modes can also be manually selected to recover electric energy and increase the driving range for electric vehicles.

### 4.2. Multi-Mode Switching Threshold Analysis

Compared with passive suspension, active suspension can better control the vehicle vibration caused by road excitation when vehicle driving conditions change. Based on the multi-mode switching type of active suspension, this is the basis of the traditional active suspension. It is more intelligent for the driver and passenger in that it automatically switches the appropriate functional mode to meet the performance requirements of different road surfaces, provide greater ride comfort, and ensure driving safety.

According to the four kinds of road switching function modules from A-grade to D-grade, a road below D-grade is rare in a normal urban setting, so they are all classified as D-grade roads.

Tire dynamic displacement directly reflects the size of the dynamic load of the tire relative to the road surface, and is an evaluation index of driving safety. Body acceleration intuitively reflects the user’s ride comfort, and is an evaluation index of car ride comfort. Therefore, these two evaluation indexes are used as evaluation criteria for mode switching, and corresponding threshold values are set.

Generally speaking, car safety is more important than comfort. Therefore, a single-dual threshold switching rule with decision order is proposed in this paper: the tire dynamic displacement is the first level of judgment criterion, the body acceleration is the second level of judgment criterion, and the conclusion is Road Mode (RM). This order determination rule makes clear the safety priority, and the efficiency of discriminant calculation is also improved.

The determination method of the suspension function module is as follows. The discrete mathematical discriminant expression is as follows:

(1) $\forall G_{q}{\left(n_{0}\right)}_{i}\leq G_{q}{\left(n_{0}\right)}_{A2}\wedge v_{i}\leq v_{t}$, ∃RM=A.

When any road identification parameter is obtained, the road power spectral density value *G_q_*(*n*_0_)*_i_* is less than the A-grade road surface limit *G_q_*(*n*_0_)*_A_*_2_ and meets the vehicle traveling speed *v_i_* ≤ *v_t_*. It can be obtained from Table 1, *G_q_*(*n*_0_)*_A_*_2_ = 32 × 10^−6^ m^−3^; when the actual value of the vertical displacement power spectral density of the driving road is less than the threshold value, and the driving speed is also less than the threshold value, the vehicle is judged to be driving on a A-grade road surface. At this time, the body acceleration and tire dynamic displacement are small, and the vehicle dynamic performance is good, so only the energy regenerative mode is needed to recover the suspension vibration energy. In addition, considering drivers’ ultimate pursuit of driving experience, this mode also provides a comprehensive mode of active suspension control for users to choose from.

Through simulation analysis and equation derivation (see Equations (4) and (5)), the RMS values of *G_q_*(*n*_0_) and *v* are proportional to the road excitation. Considering that the mean value of PSD of all levels of the road surface is divided by a geometric series with a common ratio of 4, the speed *v* is also divided by a ratio of 4 considering the ratio of high speed to low speed. In other words, the experience of driving at a higher speed on a certain level of the road surface is similar to the experience of driving at a lower speed on the next level of the road surface. After comprehensive consideration, the speed threshold *v* is selected: *v_t_* = 20 m/s. The correctness of this selection result will be explained in detail in Section 6.

(2) $\forall \left(G_{q}{\left(n_{0}\right)}_{i}\in G_{q}{\left(n_{0}\right)}_{B}\wedge v_{i}\leq v_{t}\right)$$\vee \left(G_{q}{\left(n_{0}\right)}_{i}<G_{q}{\left(n_{0}\right)}_{A2}\wedge v_{i}>v_{t}\right)$, ∃RM=B.

When the identified road parameter *G_q_*(*n*_0_)*_i_* is within the threshold range of the upper and lower limits where B-grade road is located, i.e., *G_q_*(*n*_0_)*_B_* ∈ (*G_q_*(*n*_0_)*_B_*_1_, *G_q_*(*n*_0_)*_B_*_2_] and meets the vehicle traveling speed *v_i_* ≤ *v_t_*. Among them, *G_q_*(*n*_0_)*_B_*_1_ = 32 × 10^−6^ m^−3^, *G_q_*(*n*_0_)*_B_*_2_ = 128 × 10^−6^ m^−3^. Or *G_q_*(*n*_0_)*_i_* less than the A-grade road surface limit *G_q_*(*n*_0_)*_A_*_2_ and meets the vehicle traveling speed *v_i_* > *v_t_*. At this point, the vehicle is judged to be driving on a B-grade road surface. At this time, the acceleration of the vehicle body and the dynamic displacement of the tire increase, and the ride comfort of the user decreases. The corresponding functional module is the comfort mode, which is used to improve the ride comfort of the vehicle.

(3) $\forall \left(G_{q}{\left(n_{0}\right)}_{i}\in G_{q}{\left(n_{0}\right)}_{C}\wedge v_{i}\leq v_{t}\right)$$\vee \left(G_{q}{\left(n_{0}\right)}_{i}\in G_{q}{\left(n_{0}\right)}_{B}\wedge v_{i}>v_{t}\right)$, ∃RM=C.

When the identified road parameter *G_q_*(*n*_0_)*_i_* within the threshold range of the upper and lower limits where the C-grade road is located, i.e., *G_q_*(*n*_0_)*_C_* ∈ (*G_q_*(*n*_0_)*_C_*_1_, *G_q_*(*n*_0_)*_C_*_2_] and meets the vehicle traveling speed *v_i_* ≤ *v_t_*. Where *G_q_*(*n*_0_)*_C_*_1_ = 128 × 10^−6^ m^−3^, *G_q_*(*n*_0_)*_C_*_2_ = 512 × 10^−6^ m^−3^. Or *G_q_*(*n*_0_)*_i_* falls within the upper lower limit threshold range of B-grade road, i.e., *G_q_*(*n*_0_)*_B_* ∈ (*G_q_*(*n*_0_)*_B_*_1_, *G_q_*(*n*_0_)*_B_*_2_] and meets the vehicle traveling speed *v_i_* > *v_t_*. At this point, it is judged that the vehicle is traveling on a C-grade road surface. In this condition, the dynamic displacement of the tire is large and the grip is poor. The control of vehicle suspension is more inclined to safe driving, and the corresponding function module is safety mode to improve driving safety.

(4) $\forall \left(G_{q}{\left(n_{0}\right)}_{i}>G_{q}{\left(n_{0}\right)}_{D1}\wedge v_{i}\leq v_{t}\right)$$\vee \left(G_{q}{\left(n_{0}\right)}_{i}\in G_{q}{\left(n_{0}\right)}_{C}\wedge v_{i}>v_{t}\right)$, ∃RM=D.

When the identified road parameter *G_q_*(*n*_0_)*_i_* is greater than the lower limit *G_q_*(*n*_0_) for D-grade road *G_q_*(*n*_0_)*_D_*_1_ = 512 × 10^−6^ m^−3^ and meets the vehicle traveling speed *v_i_* ≤ *v_t_*. Or *G_q_*(*n*_0_)*_i_* within the threshold range of the upper and lower limits where a C-grade road is located, i.e., *G_q_*(*n*_0_)*_C_* ∈ (*G_q_*(*n*_0_)*_C_*_1_, *G_q_*(*n*_0_)*_C_*_2_] and meets the vehicle traveling speed *v_i_* > *v_t_*. At this time, it is determined that the vehicle is driving on a road surface of grade D or above. Here, it is deemed that the road surface is D. The dynamic displacement of the tire and the acceleration of the body are large, and the dynamic performance of the vehicle becomes worse. At the same time, considering the problems of driving safety and riding comfort, the functional module selected under this working condition is the comprehensive mode, which aims to improve the comprehensive performance of the vehicle in a balanced way. In addition, the suspension vibration energy of D-grade road vehicles is high, and high speed rarely occurs, so the energy regenerative mode is provided for the driver to choose from.

According to the analysis of the above mode-switching strategy, a mode-switching rule table is developed, as shown in Table 3.

### 4.3. Analysis of Multi-Mode Switching Control Strategy

**Figure 6:**
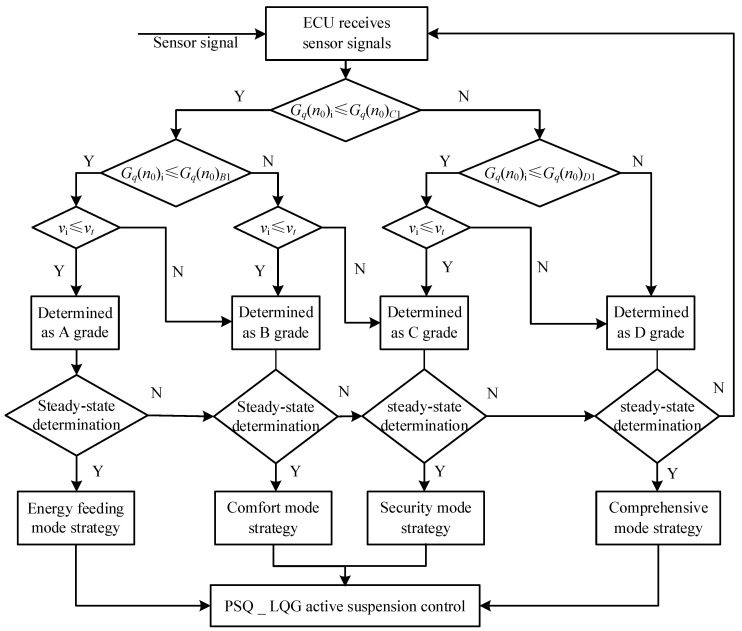
Function modules switching control flow.

The switching process of function modules is shown in Figure 6. When the road condition of the vehicle changes, the sensor on the wheel and the vehicle body transmits the signal to the ECU, and the ECU judges and processes the signal. The identification results of the road power spectral density were compared with the switching threshold. Firstly, the size of the road power spectrum was determined. After two judgments, the signal value was preliminarily divided into four numerical segments. Then according to the driving speed of the decision, we determined the state of the vehicle driving, to determine the grade of the road [38].

In this decision process, the decision frequency is the sampling frequency, and the sampling frequency of the digital acquisition card used by this project team is 200 Hz. Then, in the actual switching process, there will be the problem of excessively frequent switching. Especially in the data segment near the threshold value, the reciprocating vibration data will lead to a switch every 0.005 s under extreme working conditions. To solve this problem, steady-state judgment should be carried out to realize delayed switching. To realize delay switching and avoid the many problems caused by excessive frequent switching, such as the system response not being able to keep up and the calculations becoming complicated, the delay switching strategy is increased. Firstly, the steady-state judgment is defined as when 20 consecutive parameters are the same to determine the road surface, and the mode switch can be finally switched. The advantage of this approach is that the delay switching time is only 0.1 s, which will not cause too long a delay and make the response speed insufficient. At the same time, it can also screen out the excessively frequent switching caused by random too-large or too-small vibrations. Finally, after mode switching is completed, this information is transmitted to the suspension controller to control the suspension for mode switching.

Finally, this information is transmitted to the suspension controller to control the suspension for mode switching. For different modes, if the traditional LQR control vibration reduction is still used, the evaluation indexes of vehicle dynamic performance under various modes still cannot be effectively improved. To solve this problem, the project team seeks better and more reasonable control effects by optimizing the control coefficient of LQR in various modes, to achieve an “individualized method of instruction”, “adaption to local conditions”, and suiting “the medicine to the illness”.

## 5. Particle Swarm Optimization of the LQR Controller Design

### 5.1. Suspension LQR Controller Design

The active suspension is controlled by the LQR controller to realize the adaptive adjustment of the excitation input in the random road profile. Figure 4 shows that the LQR controller carries out real-time information acquisition through sensors, and realizes active suspension to make the active response to road excitation through active suspension control, generating corresponding force balance with it, and keeping the suspension in the optimal state.

In the design of the LQR controller, the integral value of the weighted square sum of the evaluation parameters BA, SWS, and DTD in the time domain *T* is expressed by the performance index *J*:

(18) $$J=\underset{T\to \infty }{\lim}\frac{1}{T}\int_{0}^{T}[q_{1}{\ddot{x}}_{b}{}^{2}+q_{2}{\left(x_{b}-x_{w}\right)}^{2}+q_{3}{\left(x_{w}-x_{g}\right)}^{2}]dt.$$

In the formula, *T* is the vehicle traveling time; *q*_1_, *q*_2_, and *q*_3_ are the weighting coefficients of the three evaluation parameters.

We can rewrite Equation (8) into a matrix form as follows:

(19) $$J=\underset{T\to \infty }{\lim}\frac{1}{T}\int_{0}^{T}[\begin{array}{ccc} {\ddot{x}}_{b} & x_{b}-x_{w} & x_{w}-x_{g} \end{array}]Q_{0}\left[\begin{array}{c} {\ddot{x}}_{1}\\ x_{b}-x_{w}\\ x_{w}-x_{g} \end{array}\right]dt,$$

where $Q_{0}=\left[\begin{array}{ccc} q_{1} & 0 & 0\\ 0 & q_{2} & 0\\ 0 & 0 & q_{3} \end{array}\right]$ and is substituted into Equation (9) to produce the standard quadratic form of performance index J:

(20) $$J=\underset{T\to \infty }{\lim}\frac{1}{T}\int_{0}^{T}Y^{T}Q_{0}Ydt=\underset{T\to \infty }{\lim}\frac{1}{T}\int_{0}^{T}\left(X^{T}QX+U^{T}RU+2X^{T}NU\right)dt.$$

Since the three weighting coefficients represent the relative values between the three important performance evaluation indexes in the design of the LQR controller, the weighting coefficient q of the vertical acceleration of the body is taken here for the convenience of *q*_1_ = 1, then:

$$Q=C^{T}Q_{0}C=\left[\begin{array}{ccccc} \frac{c_{s}^{2}}{m_{b}^{2}}q_{1} & -\frac{c_{s}^{2}}{m_{b}^{2}}q_{1} & \frac{c_{s}k_{s}}{m_{b}^{2}}q_{1} & -\frac{c_{s}k_{s}}{m_{b}^{2}}q_{1} & 0\\ -\frac{c_{s}^{2}}{m_{b}^{2}}q_{1} & \frac{c_{s}^{2}}{m_{b}^{2}}q_{1} & -\frac{c_{s}k_{s}}{m_{b}^{2}}q_{1} & \frac{c_{s}k_{s}}{m_{b}^{2}}q_{1} & 0\\ \frac{c_{s}k_{s}}{m_{b}^{2}}q_{1} & -\frac{c_{s}k_{s}}{m_{b}^{2}}q_{1} & \frac{k_{s}{}^{2}}{m_{b}^{2}}q_{1}+q_{2} & -\frac{k_{s}{}^{2}}{m_{b}^{2}}q_{1}-q_{2} & 0\\ -\frac{c_{s}k_{s}}{m_{b}^{2}}q_{1} & \frac{c_{s}k_{s}}{m_{b}^{2}}q_{1} & -\frac{k_{s}{}^{2}}{m_{b}^{2}}q_{1}-q_{2} & \frac{k_{s}{}^{2}}{m_{b}^{2}}q_{1}+q_{2}+q_{3} & -q_{3}\\ 0 & 0 & 0 & -q_{3} & q_{3} \end{array}\right];\;N=C^{T}Q_{0}D=\left[\begin{array}{c} -\frac{c_{s}}{m_{b}^{2}}q_{1}\\ \frac{c_{s}}{m_{b}^{2}}q_{1}\\ -\frac{k_{s}}{m_{b}^{2}}q_{1}\\ \frac{k_{s}}{m_{b}^{2}}q_{1}\\ 0 \end{array}\right];\;R=D^{T}Q_{0}D=\frac{1}{m_{b}^{2}}$$

According to the optimal control theory, the optimal control force can be obtained:

(21) F=−KX,

where *K* is the optimal state feedback matrix, $K=R^{-1}\left(B^{T}P+N^{T}\right)$, which is determined by vehicle parameters and weighting coefficient. When the vehicle parameter value and the weighting coefficient value are determined, *K* can be calculated by the Riccati equation and can be expressed as follows:

(22) $$PA+A^{T}P-\left(PB+N\right)R^{-1}\left(B^{T}P+N^{T}\right)+Q=0.$$

### 5.2. Particle Swarm Optimization Algorithm

Particle swarm optimization is a heuristic global optimization algorithm based on animal group foraging and human decision-making behavior [46]. In this algorithm, the particles pass through the individual extremum *P_t_* and the population extremum *G_t_* updates its speed *V* and position *X*, whose evolutionary equation can be described as follows:

(23) $$V_{t+1}=mV_{t}+c_{1}r_{1}\left(P_{t}-X_{t}\right)+c_{2}r_{2}\left(P_{t}-X_{t}\right)$$

(24) $$X_{t+1}=X_{t}+V_{t+1},$$

where *m* is the weight of inertia; *c*_1_, *c*_2_ update parameters for speed; and *r*_1_, *r*_2_ are random numbers in the range [0, 1].

To better balance the global search and local search capability of the algorithm, we can adopt a linearly decreasing inertia weight:

(25) $$w=w_{\mathrm{start}}-\frac{t\left(w_{\mathrm{start}}-w_{\mathrm{end}}\right)}{T},$$

where *w*_start_, *w*_end_ represent the initial inertia weight and the inertia weight when iterating to the maximum number, resepectively; *t* is the current number of iterations and *T* is the maximum number of iterations.

Generally speaking, the initial inertia weight is greater than the inertia weight when the number of iterations is maximum so that the particle swarm optimization algorithm can maintain a strong global search ability with a larger inertia weight in the early iteration, and carry out a more accurate local search with a smaller inertia weight in the later iteration. However, with the increasing number of iterations of the particle swarm optimization algorithm, the particles will become more and more similar, and it is easy to fall into the local minimum and be unable to jump out. Therefore, the crossover and mutation operation of the particle swarm optimization algorithm is introduced to search for the optimal solution by crossing the individual particle and the group extreme value and the particle’s variation.

Crossover: Because the particle swarm individual uses real number coding, the crossover operation uses the real number crossover method, the *m* chromosome *A_m_,* and the *n* population optimal chromosome *A_n_*; the method of crossover operation at the *k* position is:

(26) $$\{\begin{array}{c} A_{mk}=rA_{mk}+\left(1-r\right)A_{nk}\\ A_{nk}=\left(1-r\right)A_{mk}+rA_{nk} \end{array},$$

where *r* is a random number in the interval [0, 1].

Variation: The main purpose of a variation operation is to maintain the diversity of the population. Mutation operation randomly selects an individual from the population and selects one point of the individual for the mutation to produce a better individual. The operation method of mutation of the *j*th gene of the *i*th individual is:

(27) $$A_{ij}=\{\begin{array}{c} A_{ij}+\left(A_{ij}-A_{\max}\right)f\left(t\right),q\geq q_{0}\\ A_{ij}+\left(A_{\min}-A_{ij}\right)f\left(t\right),q<q_{0} \end{array}$$

(28) $$f\left(t\right)=1-r^{{\left(1-t/T\right)}^{a}},$$

where *A_max_* and *A_min_* are the upper and lower bounds of individual *A_ij_*; *r* is a random number in the interval [0, 1], *t* is the current evolutionary algebra, *T* is the maximum evolutionary algebra, and *a* is a tunable parameter.

**Figure 7:**
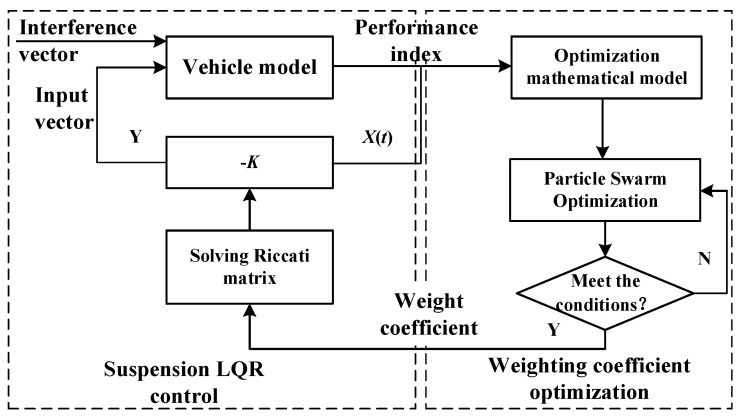
LQR controller optimization process of particle swarm optimization algorithm.

The optimization process can be visually described as shown in Figure 7.

Due to the inconsistency of units and orders of magnitude in performance indicators such as vertical acceleration of the vehicle body, dynamic travel of suspension, and deformation of the roadwheel rubber ring, a fitness function was designed by dividing the RMS value of the corresponding index of active suspension by the RMS value of the corresponding index of passive suspension. The fitness function is as follows:

(29) $$L_{\min}=\frac{BA\left(X\right)}{BA_{P}}+\frac{SWS\left(X\right)}{SWS_{P}}+\frac{DTD\left(X\right)}{DTD_{P}},$$

where $X=\left(\begin{array}{llll} q_{1} & q_{2} & q_{3} & r \end{array}\right)$; *r* = 1; 0.1 < $q_{i}$ < 10^8^; *i* = 1, 2, 3; *BA*(*X*), *SWS*(*X*), and *DTD*(*X*) respectively represent the active and passive RMS values of body vertical acceleration, suspension dynamic travel, and wheel dynamic displacement.

**Figure 8:**
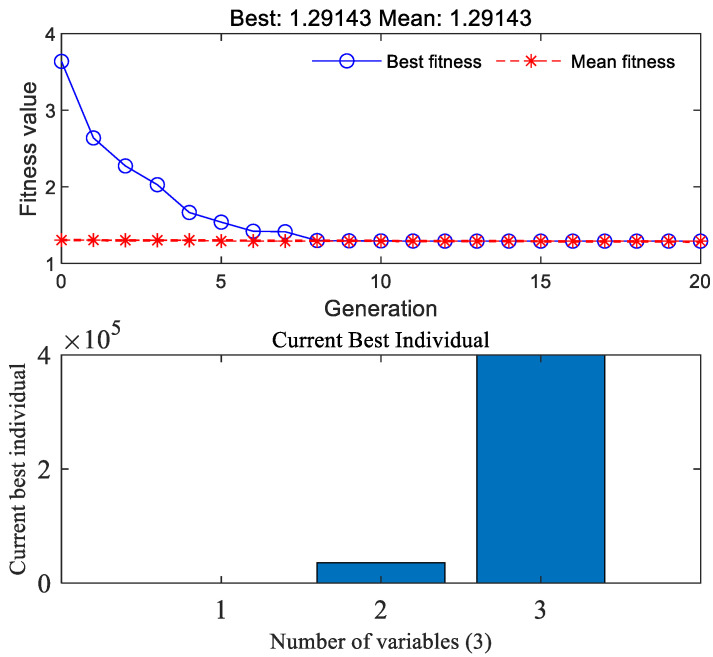
Iterative optimization results of genetic algorithm.

After solving the RMS values of suspension performance evaluation parameters under each road grade from A to D, we can input the RMS values of the above passive suspension into Equation (29), and use a genetic algorithm to iteratively search for optimization, as shown in Figure 8. The LQR weight coefficient q of optimizing based on the genetic algorithm is obtained under different roads *q*_1_, *q*_2_, and *q*_3_, as shown in Table 4. After obtaining the road grade estimate, the dynamic characteristics existing in the suspension switching system are classified according to the speed. Finally, through the weight coefficient shown in Table 4, the optimal value is found again to improve the vehicle operating conditions.

## 6. Simulation and Test Verification

### 6.1. Road Grade Identification Test

In this section we establish a test system for the test vehicle, collect acceleration data transmitted from road unevenness to body vibration through acceleration sensors, and analyze and verify the results.

**Figure 9:**
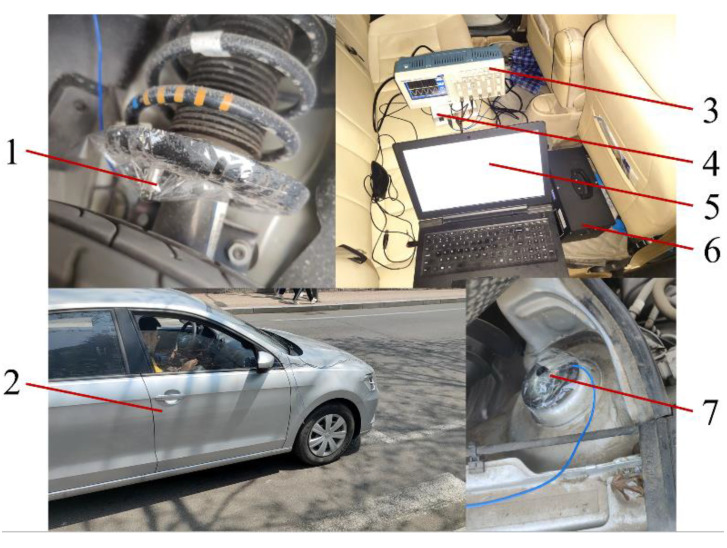
Test vehicle and test equipment diagram. 1/7. Acceleration sensor 2. Test car 3. Digital oscilloscope 4. Data acquisition card 5. Upper computer 6. External power supply.

Figure 9 shows the installation position and driving scene of the test equipment in the real vehicle road test. The test vehicle used by the project team had independent suspension modified by Volkswagen Lavida. Two acceleration sensors were placed on the lower end of the suspension of the left front wheel of the test vehicle. The upper end of the sensor collected vibration signals from the body, and the lower end of the sensor collected vibration signals from the vehicle chassis and suspension system close to the hub. The sensor model used was PCB 35C33, and the sampling sensitivity was 100 mV/g. At the same time, a four-channel data acquisition card was used to convert the sensor signal, and the sampling frequency was 200 Hz. A digital oscilloscope was used for data acquisition and recording, and the final data processing was completed by computing the force analysis provided by the upper computer.

**Figure 10:**
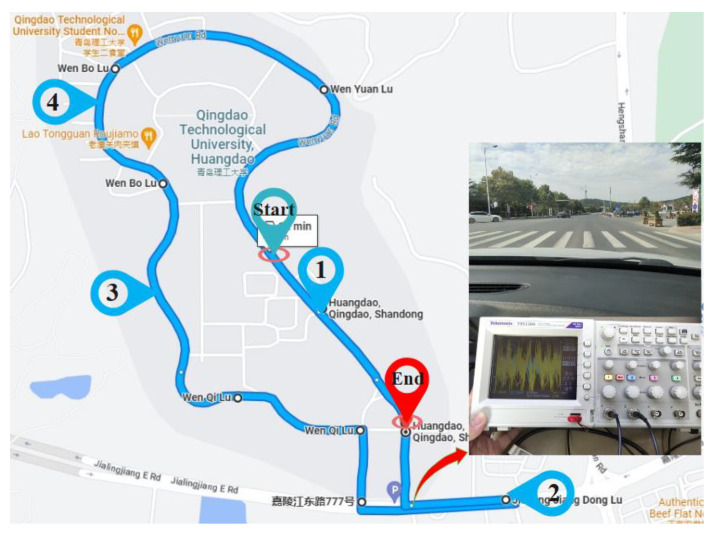
Test route plan for the test vehicle.

After the completion of the equipment construction, we drove the test vehicle to the designated road test section for testing, as shown in Figure 10. The test vehicle collected vibration signals at different road sections and speeds to obtain real road excitation signal data. The test section was divided into four road sections. At the same time, to ensure the relative accuracy of data, when testing the same speed, they all drove on the same section of road, in the same lane, and maintained speed for five reciprocating drives. In Section 1, several groups of six different speeds of 10–60 km/h were tested. In Section 2, multiple sets of tests were carried out at speeds of 18 km/h (5 m/s), 36 km/h (10 m/s), and 72 km/h (20 m/s). For roads 3 and 4, due to road restrictions, only three groups at 10 km/h, 20 km/h, and 30 km/h were tested.

To verify the accuracy of the road estimation algorithm for road solution, the project team used a traditional three-meter engineering measuring ruler to manually measure the road profile (curve) unsmooth ness function *q*(*I*)—that is, the elevation *q* of the road relative to the reference plane and the change in length I along the road strike. The measured unevenness function was processed, the signal was decomposed into a series of Fourier components, and a linear spectrum was generated by a Fourier transform and expressed in double logarithmic coordinates. Finally, the power spectral density was used to replace the spectral line, and the road roughness coefficient *G_q_*(*n*_0_) was obtained.

**Figure 11:**
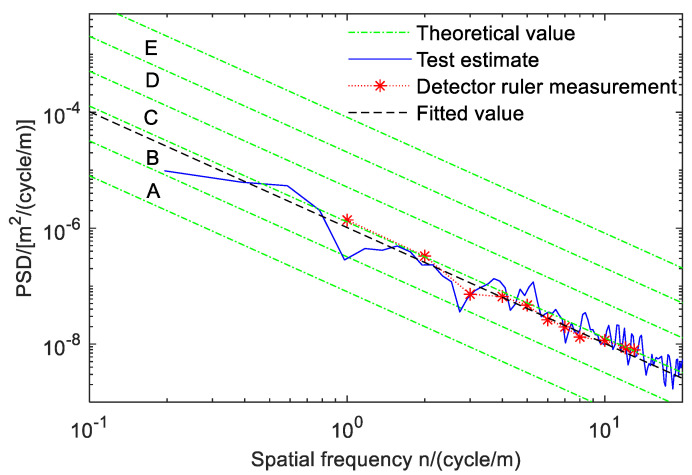
Comparison of theoretical and test values of road PSD.

The upper and lower limits of A–E grade road power spectral density in Table 1 and the road Section 1 in Figure 10 were solved by the minimum estimation square estimation criterion, and the processed data measured by multiple groups with a traditional 3-m detection ruler were compared with the fitted road power spectral density. The results are shown in Figure 11. The blue-green dotted lines are the upper and lower limits of the A–E grade, while the ranges of all levels of the road surface are between the upper and lower limits, which have been marked in the figure. The blue solid line is the PSD value of road section 1 obtained by the inverse solution of the sensor signal through the road estimation algorithm. The red dotted line matched with the asterisk is the measurement processing data of the 3-m measuring ruler. Finally, the maximum gap data of the pile in the road measuring point distance measured by the measuring ruler was fitted, and the data of the black dotted line were obtained. Among them, the fitting PSD value was *G_q_*(*n*_0_)*_f_* = 108 × 10^−6^ m^3^ at the reference spatial frequency *n*_0_ of road section 1. It can be seen from the figure that the PSD value obtained by the road estimation method is similar to the measured value of the three-meter detector, which verifies the feasibility and correctness of the method.

**Figure 12:**
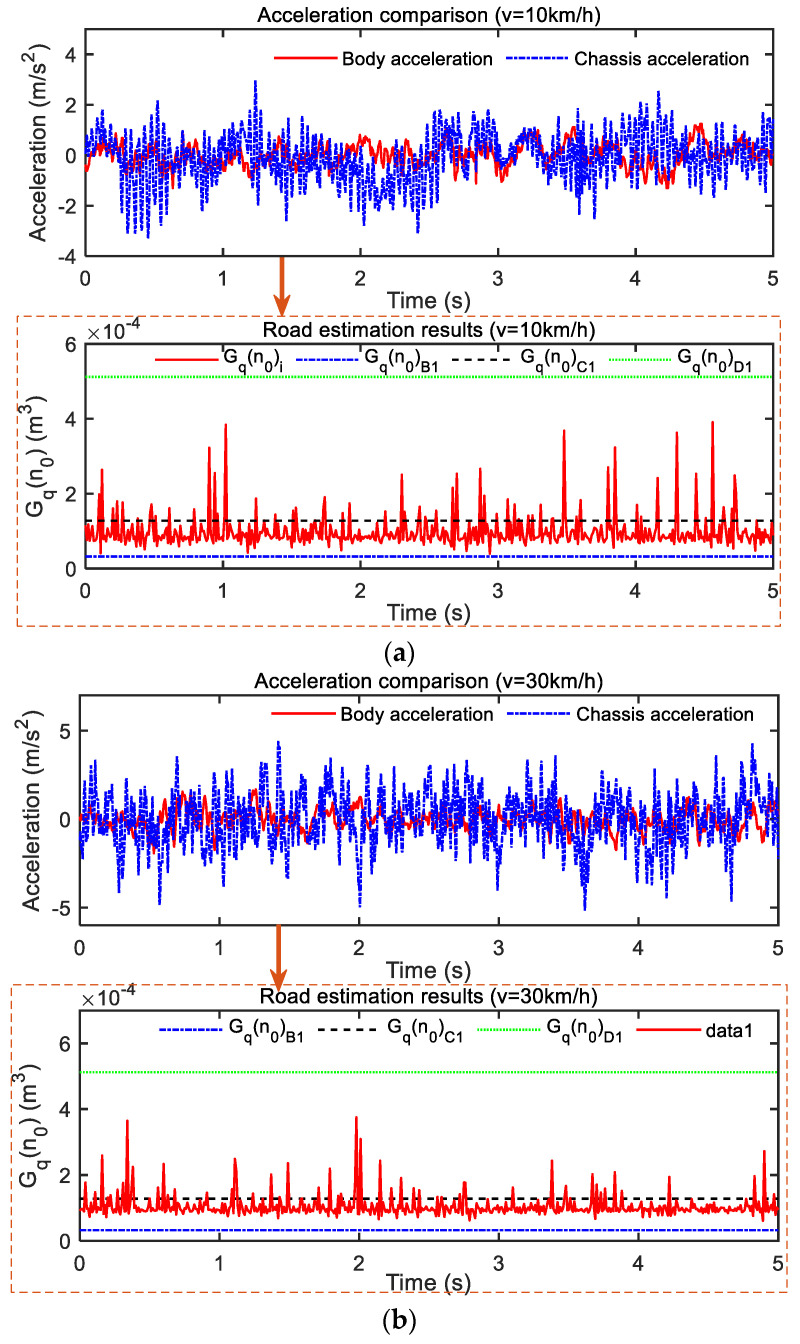

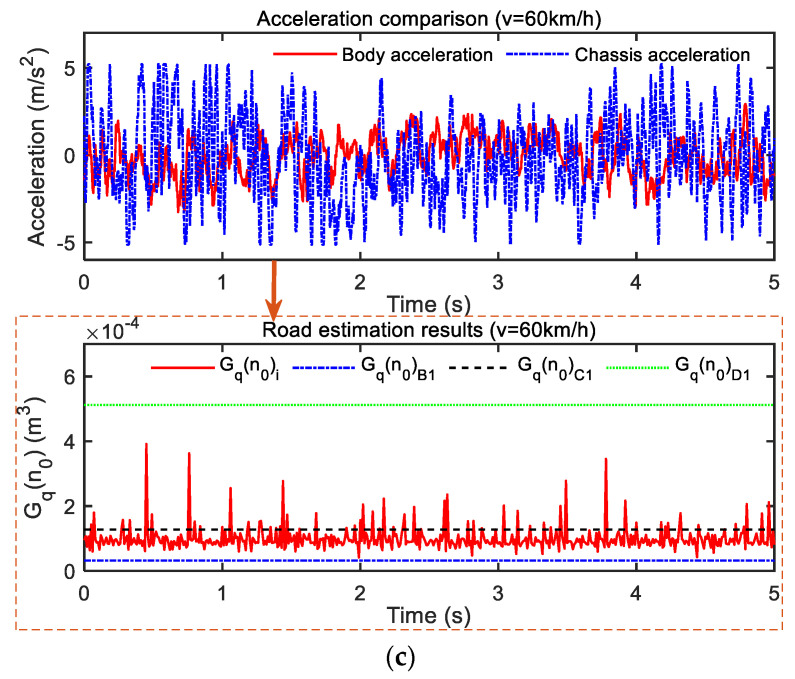
Comparison of road estimation under three speeds in road section 1. (**a**) Comparison of results when *v* = 10 km/h. (**b**) Comparison of results when *v* = 30 km/h. (**c**) Comparison of results when *v* = 60 km/h.

To further verify the correctness of the test results, three different speeds of vehicles in road section 1 were analyzed. Figure 12a–c show the road PSD value obtained by reverse estimation of chassis acceleration and body acceleration collected by a sensor under a traveling speed of 10 km/h, 30 km/h and 60 km/h, respectively. As can be seen from Figure 12a, chassis acceleration was significantly greater than body acceleration at a traveling speed of 10 km/h, which is caused by the suspension’s blocking and consumption of vibration. The vibration of body acceleration was always maintained at ±1.5 m/s^2^, while the vibration of chassis acceleration was maintained at ±3 m/s^2^ between. The road estimation result by the least squares method *G_q_*(*n*_0_) was kept at the lower limit of the C-grade road surface represented by the dotted line (also the upper limit of the B-grade road surface) *G_q_*(*n*_0_)*_C_*_1_ nearby. When the speed increased to 30 km/h, as shown in Figure 12b, compared with Figure 12a, the amplitude of acceleration increased somewhat, but the PSD value estimated by the road surface did not increase or decrease significantly. This is because, when the speed increases, the driving vehicle’s excitation from the road also increases. However, since the test road section is always the same, the road estimation results remain unchanged. This also verifies the correctness of the road surface estimation method. The vibration of its body acceleration is kept at ±2.5 m/s^2^, while the vibration of chassis acceleration is maintained at ±5 m/s^2^ between. While the road estimation result by the least squares method *G_q_*(*n*_0_) is the same as in Figure 12a, which stays in the lower limit *G_q_*(*n*_0_) of C-grade road represented by the dotted line *G_q_*(*n*_0_)*_C_*_1_ nearby. When we continue to increase the speed to 60 km/h, as shown in Figure 12c, the body acceleration and chassis acceleration values continue to increase, while the road surface estimate remains similar to that in Figure 12a,b.

To analyze the results quantitatively, the acceleration values and road estimates under three different speeds in Section 1 were processed by root-mean-square processing, and the results are shown in Table 5. It can be seen that, in road section 1, the RMS values of body acceleration and chassis acceleration at three different speeds increased with an increase in driving speed: 0.4512 m/s^2^ and 0.9919 m/s^2^ at 10 km/h, 0.5715 m/s^2^ and 1.5667 m/s^2^ at 30 km/h, and 1.1337 m/s^2^ and 2.4482 m/s^2^ at 60 km/h, respectively. However, the value of *G_q_*(*n*_0_) was the same: 108.84 × 10^−6^ m^3^ at 10 km/h, 110.14 × 10^−6^ m^3^ at 30 km/h, and 106.98 × 10^−6^ m^3^ at 60 km/h. This is very close to the result of 108.84 × 10^−6^ m^3^ obtained by the 3-m detection ruler method, and the comprehensive error was less than 2%. This can prove the feasibility, correctness, and accuracy of the road estimation method.

The comprehensive error results were compared with [27,28], and the tire profile values were estimated by the combination of AKF, RM algorithm, and RTS smoothing and optimized by GA. In the PSD and IRI estimation of six different road sections of 9 km, the maximum average error did not exceed 8%. The estimation model proposed in this paper has the advantage of a lower error rate than its model.

**Figure 13:**
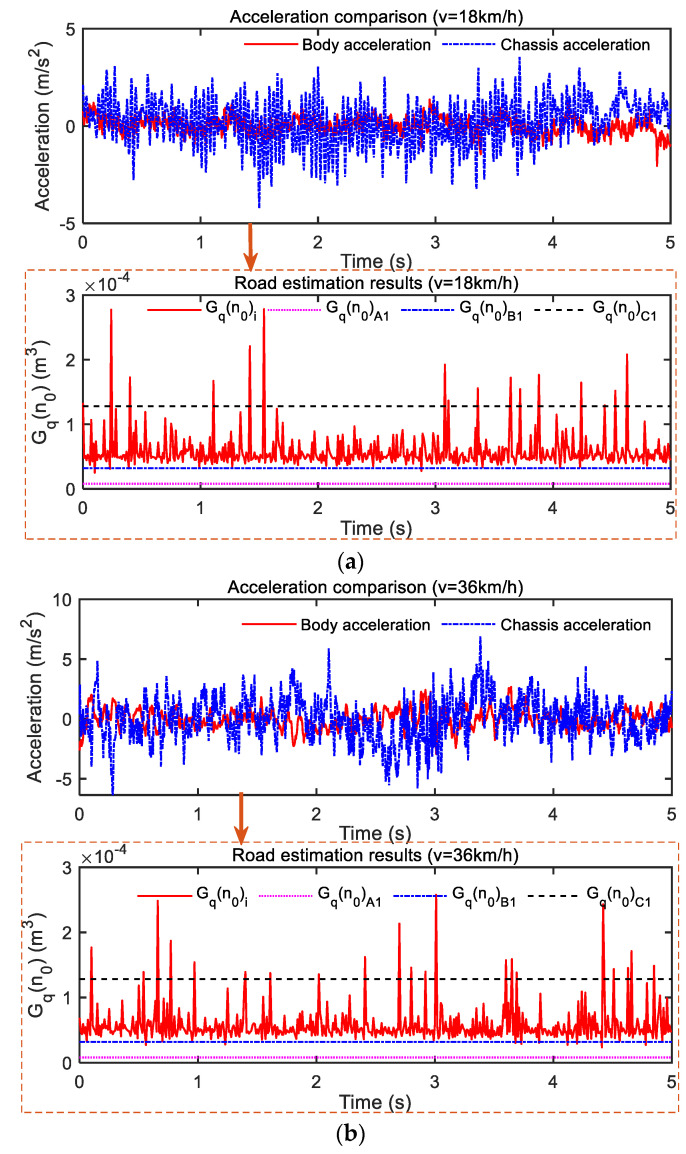

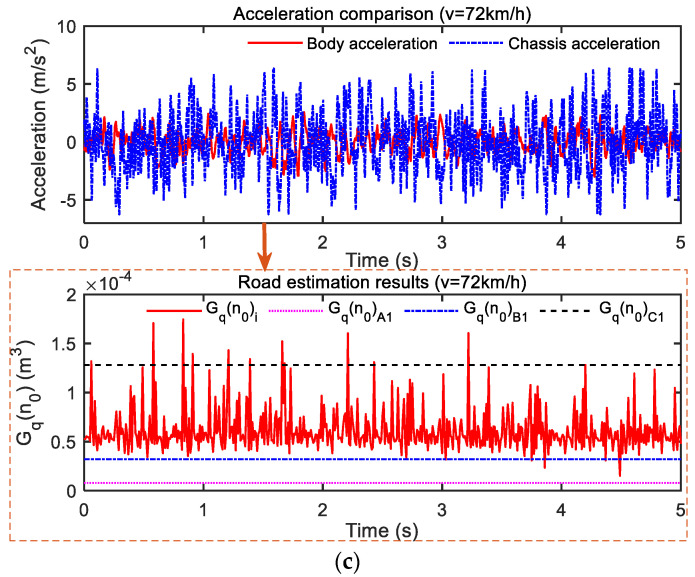
Comparison of road surface estimation at three speeds in road section 2. (**a**) Comparison of results when *v* = 18 km/h. (**b**) Comparison of results when *v* = 36 km/h. (**c**) Comparison of results when *v* = 72 km/h.

Data signals of vehicle driving excitation under three different speeds were also collected for road section 2, and the test results are shown in Figure 13. The three different speeds were 18 km/h (5 m/s), 36 km/h (10 m/s), and 72 km/h (20 m/s). As can be seen from Figure 13a, the vibration of the body acceleration was maintained at ±2 m/s^2^ between, while the vibration of chassis acceleration was maintained at ±4 m/s^2^ between. When the speed increased to 36 km/h, as shown in Figure 13b, the amplitude of acceleration increased compared with that in Figure 13a, which was caused by an increase in speed. At this speed, the vibration of the acceleration of the body was maintained at ±3 m/s^2^ while the vibration of chassis acceleration was maintained at ±5 m/s^2^ between. When we continued to increase the speed to 72 km/h, as shown in Figure 13c, the acceleration of the body and chassis continued to increase, and the vibration amplitude of both was maintained at ±4 m/s^2^ and ±6 m/s^2^ between. Looking again at the road estimates of Figure 13a–c through the least squares road estimate, the result *G_q_*(*n*_0_) was consistent at the three different vehicle speeds, and stayed at the lower limit *G_q_*(*n*_0_) of B-grade road surface represented by the dotted line G_q_(n_0_)*_B_*_1_; the dashed line represents the B-grade road limit *G_q_*(*n*_0_)*_B_*_2_ (also the lower limit of C-grade road *G_q_*(*n*_0_)*_C_*_1_) nearby.

The test results at three different speeds in Section 2 were treated with root-mean-square processing for quantitative analysis. The results are shown in Table 3. It can be seen that the RMS values of body acceleration and chassis acceleration under three different speeds in road section 2 increased with the increase in driving speed. *G_q_*(*n*_0_), 66.92 × 10^−6^ m^3^ at 18 km/h, respectively, 65.94 × 10^−6^ m^3^ at 3 km/h, 65.39 × 10^−6^ m^3^ at 72 km/h. It can be proven that the road estimation method can decouple the influence of vehicle speed on vehicle vibration well. Although the vibration signal collected by the sensor is affected by the driving speed, the speed can be decoupled under the same road section, and the solution of the power spectral density value of the road profile in the pure spatial domain can be realized.

It is also noted that the RMS values of BA and CA at 72 km/h are 0.9487 m/s^2^ and 2.5729 m/s^2^, while the RMS value of BA and CA at 36 km/h are about $\sqrt{2}$ times 0.7764 m/s and 1.7760 m/s^2^, respectively. The RMS values of BA and CA at 36 km/h are about $\sqrt{2}$ times 0.4417 m/s at 18 km/h, respectively. This is no coincidence; because $\sqrt{72/36}=\sqrt{36/18}=\sqrt{2}$ from Equation (4), it can be concluded that the road excitation is correlated with *G_q_*(*n*_0_) and is proportional to the square root of *v*. (See Table 1.) Again, the average value of each adjacent road grade is a geometric series, and its common ratio is 4. The following conclusion can be obtained by connecting the road excitation with the square root of *G_q_*(*n*_0_).

In theory, the road excitation value of driving at 72 km/h on a B-grade road has the same effect as driving at 18 km/h on a C-grade road; 18 km/h represents a lower speed drive, 36 km/h represents a moderate speed drive, and 72 km/h represents a higher speed drive. In other words, the experience of driving at higher speeds on one level of the road surface is similar to the experience of driving at lower speeds on the next level. This also proves that the selection of threshold *v_t_* in Section 4 is reasonable.

### 6.2. Multi-Mode Switching Control Strategy Test

**Figure 14:**
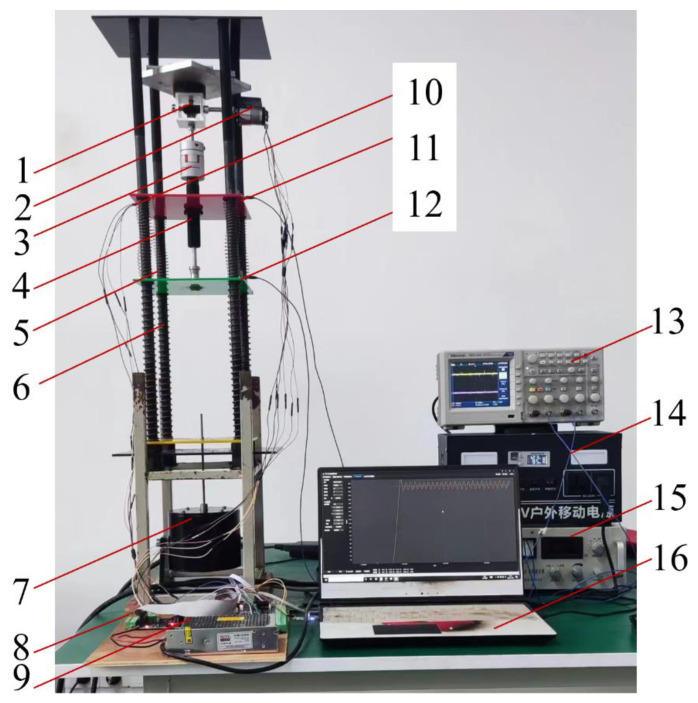
Test diagram of mode switching control on the variable road surface. 1. Gear mechanism 2. DC motor 3. Coupling 4. AD adjustable damper 5/6. Spring 7. Actuator 8. Controller 9. Data pickup card 10. Feedback acceleration sensor 11/12. The acceleration sensor 13. Oscilloscope 14. DC regulated power supply 15. Power Amplifier 16. Upper computer.

To verify the control effect of switching modes under different road surfaces, the project team built a quarter-vehicle model test bench with two degrees of freedom, as shown in Figure 14 [47,48]. The controller is used to control the speed and rotation direction of the motor, and the motor changes the spatial transmission direction through the gear mechanism to realize the reciprocating motion control of the active suspension. At the same time, the power is transmitted by the motor through the gear and coupling to the damping adjustment knob of the AD adjustable damper, which can also realize the adjustment of different damping values. The feedback acceleration sensor transmits the signal through the controller to the supreme position machine and realizes the output control feedback of the active suspension. The counterweight block above the gear mechanism simulates the body mass *m_b_*. The other modules, since they are mounted on dampers, do not include mass. Below the damper, the green acrylic plate before springs 5 and 6 simulates the tire mass *m_w_*. Spring 5 represents suspension stiffness *k_s_* and spring 6 is for tire stiffness *k_t_.* The bottom of the actuator simulates the road excitation of the vehicle driving under different roads; the control of the actuator to achieve different inputs is by the power amplifier and the upper computer. The power amplifier output adjustment signal to the actuator achieves the simulation of different road surface excitation. Sensors 10 and 11 collect the vibration signals of the upper and lower parts of the damper, respectively, and the vibration signals are analyzed by the oscilloscope through the data acquisition card [5].

Due to the equipment limitation of the test bench, 1:1 reproduction was not carried out completely according to the automobile body parameters; the numerical value of the parameters was reduced based on the original body parameters. The actual test parameters of the specific test bench are shown in Table 6.

To verify the rationality of different road mode switching, the power amplifier was output to the actuator with different amplitudes of sinusoidal excitation. To imitate the vehicle driving under different road conditions, vibration signals were collected by sensors to compare the passive suspension, traditional active suspension control, and active control under the mode switching strategy. First of all, the test bench vibration signal was collected in a completely passive state when the DC motor was not working. Secondly, the vibration signal under the traditional LQR control was collected when the DC motor was working (weighted coefficient *q*_1_ = 1, *q*_2_ = 5, *q*_3_ = 80,000). Finally, the vibration signal under LQR control based on PSO optimization was collected, and its weighting coefficient is shown in Table 4.

Sinusoidal road excitation equations of different amplitudes output to the actuator are shown below, where frequency *f* = 5 Hz.

(30) $$x_{g}\left(t\right)=\{\begin{array}{c} 0.01\sin\left(2\pi ft\right)\;t\in \left[0s,3s\right)\\ 0.015\sin\left(2\pi ft\right)\;t\in \left[3s,6s\right] \end{array}$$

**Figure 15:**
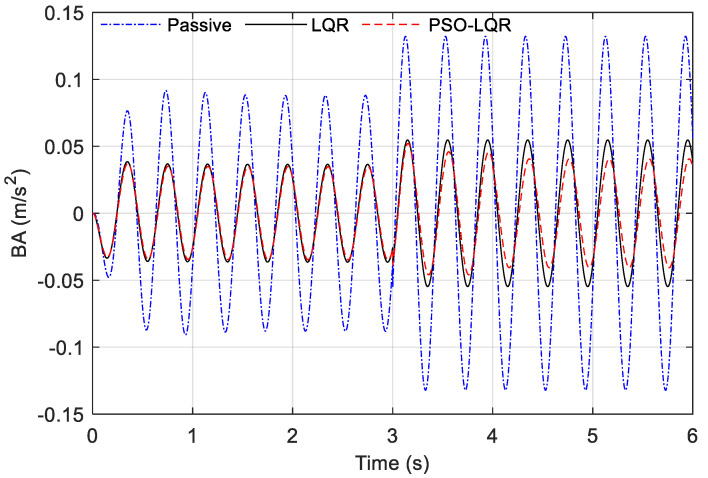
Comparison of mode switching control tests under sinusoidal excitation.

We compared the three groups of body acceleration data collected, and the results are shown in Figure 15. It can be seen that in the vibration process of 0 to 3 s, the acceleration amplitude of the passive suspension represented by the blue dotted line was the largest, far exceeding the two groups of data of the active suspension. The red dotted line represents the LQR control optimized based on PSO, and its value was slightly less than the vibration amplitude of the traditional LQR control represented by the black solid line. At 3 s, the vibration amplitude of the actuator changed from 0.01 m to 0.015 m, while the acceleration amplitude of the passive suspension continued to increase, and the acceleration change was ±0.14 m/s^2^ between. Based on PSO_LQR controls switch to the control strategy in comfort mode, the control effect under the new weight coefficient optimization was better than the traditional LQR control effect. It can be seen from the figure that the BA vibration amplitude of PSO_LQR was kept within the range of less than ±0.05 m/s^2^. The BA amplitude of traditional LQR was kept in the range slightly higher than ±0.05 m/s^2^.

The data in Figure 15 were quantitatively analyzed, and the results are shown in Table 7. It can be seen that the RMS values of BA of the passive suspension in the two time periods were 0.0604 m/s^2^ and 0.0934 m/s^2^, respectively. Compared with the passive suspension, the vibration amplitude of both active suspensions was at least 55% higher. Of course, this boost comes at a cost, in the form of extra energy consumed. Especially at 3 s to 6 s, the PSO_LQR’s BA RMS value was optimized by 21.45% compared to the traditional LQR. Through the test bench of the 1/4 vehicle model, we proved that the mode-switching control strategy based on the estimation of road roughness can provide better driving comfort compared with the single control of the traditional LQR. Based on the above test results, the project team conducted a simulation analysis of suspension control under mode switching based on road surface estimation, and the vehicle parameters used are shown in Table 2. The following working conditions were constructed for analysis. The vehicle was driving at a speed of 60 km/h on the A-level road surface, and the road surface changed once every 5 s. The road surface was successively A, C, B, and D.

**Figure 16:**
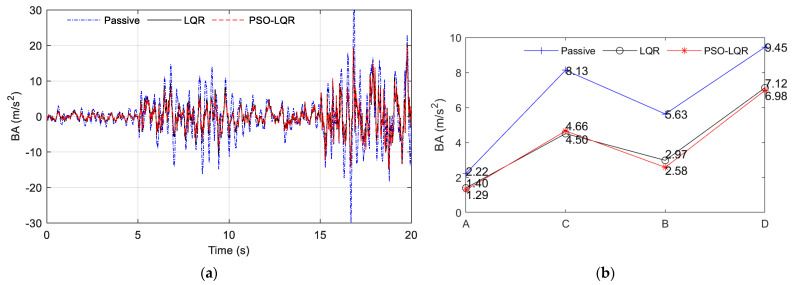
BA comparison diagram of mode switching on the changed road surface. (**a**) Time domain variation curve of A–C–B–D road section. (**b**) RMS value of A–C–B–D road section.

**Figure 17:**
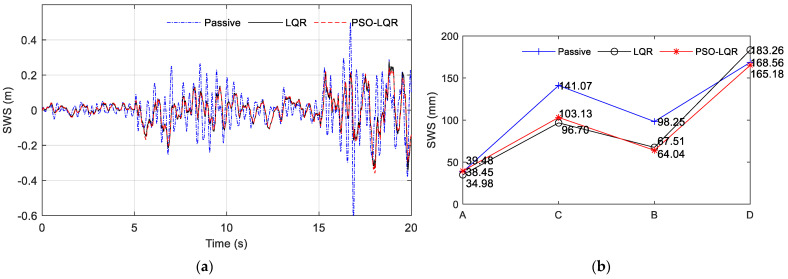
SWS comparison diagram of mode switching on the variable road surface. (**a**) Time domain variation curve of A–C–B–D road section. (**b**) RMS value of A–C–B–D road section.

**Figure 18:**
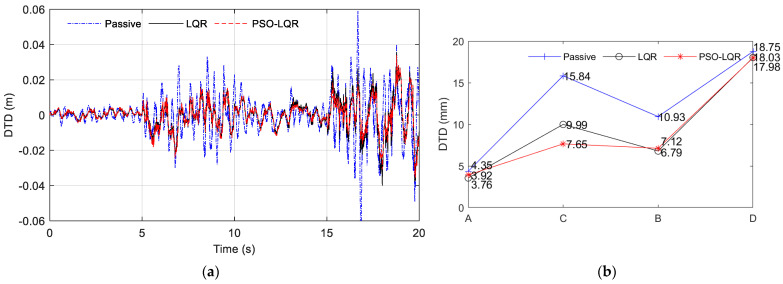
DTD comparison diagram of mode switching on the variable road surface. (**a**) Time domain variation curve of A–C–B–D road section. (**b**) RMS value of A–C–B–D road section.

Through the analysis of specific working conditions on the abrupt road part of the A–C–B–D road section, the comparison results of vehicle dynamic performance evaluation indexes BA, SWS, and DTD were obtained, as shown in Figure 16, Figure 17 and Figure 18, respectively. As shown in subfigure (a) of Figure 16, Figure 17 and Figure 18, PSO_LQR optimization control is represented by a red dotted line, while traditional LQR control is represented by a black solid line, and passive suspension is represented by a blue dotted line that serves as a control group. As shown in subfigure (b) of Figure 16, Figure 17 and Figure 18, the comparison result of passive suspension is represented by a blue plus, traditional LQR control is represented by a black circle, and the quantitative index of active suspension based on PSO_LGR optimization control is represented by a red asterisk. It can be seen that, in the whole process, the BA and DTD of the passive suspension were worse than those of the two active suspensions, which indicates that the active suspension is better in terms of ride comfort and handling. In the face of sudden changes in the road surface, compared with the traditional LQR control, the PSO_LGR optimization control strategy based on the road roughness and other parameters can quickly follow the mutation of the vehicle, and improve the ride comfort or maintain the handling stability to be more targeted for different road conditions.

The results of the three subgraphs (a) of Figure 16, Figure 17 and Figure 18 are analyzed; 0–5 s refers to the vehicle driving at 60 km/h on the A-grade road surface. To reflect the comparison of the three suspension types, the PSO_LQR control here switched to the comprehensive mode, pursuing a higher driving experience, rather than the energy regenerative mode under the passive suspension. The BA vibration amplitude of passive suspension was greater than that of active suspension controlled by LQR and PSO_LQR. At the same time, the BA values of traditional LQR and PSO_LQR were the same, within ±4 m/s^2^ between. The travel of SWS was also within the allowable vibration travel range of suspension. The change of DTD was similar to the situation of BA. It can be seen from the quantitative index RMS value that the BA optimization of active suspension controlled by PSO_LQR was 41.89% compared with that of passive suspension and 7.86% compared with that of LQR suspension. In the evaluation index of DTD, PSO_LQR was 13.56% higher than passive suspension and 4.08% lower than LQR. The PSO_LQR control was more reasonable than the traditional LQR control to greatly improve the riding comfort with less loss of handling stability.

At 5 s, the road changed from an A-grade to a C-grade road. During this period, the amplitude of passive suspension BA is large, indicating the worst comfort. The DTD value of PSO_LQR was smaller than that of the traditional LQR, which proves that the weight coefficient of the controller changed, thus improving the driving safety index. This is also consistent with the corresponding switching mode under the C-grade road surface, which pays more attention to the stability and safety of the handling. At this time, the control strategy is actually to sacrifice riding comfort to improve driving safety, because the output of the main power is certain. At the same time, improving BA and DTD goes against the law of energy conservation, which is unrealistic. On a C-grade road surface, the PSO_LQR control and LQR control both had a BA value of ±10 m/s^2^. However, in a quantitative index analysis, with PSO_LQR control for this period compared with passive suspension and LQR control, the DTD value increased by 51.70% and 23.42%, respectively. In the quantization index of BA, PSO_LQR control was 3.43% lower than LQR control in DTD value. This indicates that PSO_LQR control, in safety mode, sacrifices a small amount of comfort in exchange for a large increase in handling safety.

After 10 s, the road level changed from C-grade to B-grade. According to the control strategy of mode switching, this mode is called comfort mode. First, it can be seen that the BA amplitude under passive suspension was still larger than that under LQR control and PSO_LQR control of the active suspension. With the control strategy switching of PSO_LQR, the values of BA and DTD tended to be similar to those of LQR. Quantitative analysis showed that the BA optimization of PSO_LQR controlled active suspension was 54.17% better than that of passive suspension, and 13.13% better than that of LQR suspension. Among the evaluation indexes of DTD, PSO_LQR was 34.86% and 4.36% better than passive and LQR, respectively. From these two quantitative indicators, the comfort mode of the control on the B-grade road surface can better realize the requirements of driving comfort.

After 15 s, the road level changed from B to D. This mode is a comprehensive mode, which is similar to the previous 5 s condition. It should be pointed out that this is only the optimization effect based on this kind of control strategy under this particular working condition, and different working conditions have different emphases on optimization. The control strategy proposed in this paper aims to achieve a more comprehensive control effect according to the changes in vehicle driving conditions, and improve the driving experience more intelligently and comprehensively.

## 7. Conclusions

(1) In this paper, a road input model of varying road surface and speed was constructed theoretically, and the road excitation of different road surfaces was simulated by combining the state space equation of the automobile suspension system. Through the simulation verification, the road excitation *X_g_* was the coupling between the grading coefficient of different roads and the vehicle speed and other parameters.
(2) Through the establishment of a improved least squares road model, a sensor was used to collect the sprung and unsprung acceleration of the vehicle suspension system. The road excitation and decoupling speed, which are difficult to collect by a sensor, were estimated in reverse, and the road elevation information in the pure spatial domain was obtained. According to the road test of the test vehicle, the estimated value of the road surface under three different speeds in Section 1 was, respectively, 108.84 × 10^−6^ m^3^ at 10 km/h, 110.14 × 10^−6^ m^3^ at 30 km/h, and 106.98 × 10^−6^ m^3^ at 60 km/h. The results were very close to the 108.84 × 10^−6^ m^3^ obtained by the measuring ruler method, and the comprehensive error was less than 2%.
(3) According to the solution results of road estimation, threshold values under different roads and speeds were divided. Four different control switching modes of the suspension system were established based on the theory of “suit the medicine to the illness.” A particle swarm optimization algorithm was used to optimize the weight coefficients of LQR control, and the weight coefficients of different mode-switching strategies were solved. At the same time, a steady-state model was constructed to judge the switching process to avoid the excessive response caused by frequent switching.
(4) Through the construction of a quarter of the vehicle suspension system model test bench, it was proven that, under the mode switching control strategy, compared with the passive suspension and the traditional single control of LQR, the mode switching control strategy based on the estimation of road roughness could provide better ride comfort. The simulation and test results also showed that, when the road changed from A-grade to C-grade and the mode switched to safe mode, the PSO_LQR control improved the DTD value by 51.70% and 23.42%, respectively, compared with passive suspension and LQR control, indicating that the control safety and stability were improved. When a C-grade road changed to a B-grade road, the BA optimization of PSO_LQR controlled active suspension was 54.17% better than that of passive suspension, and 13.13% better than that of LQR suspension, which indicates that the driving comfort was improved. In the comprehensive mode, the dynamic performance evaluation index was better. Therefore, we proved that the multi-mode switching control strategy proposed in this paper can achieve a more comprehensive control effect according to the changes in vehicle driving conditions. From the perspective of quantitative indicators, multi-mode switching based on road surface changes can better achieve a balance between riding comfort and handling safety and stability, and this strategy also improves the driving experience more intelligently and comprehensively.

## Figures and Tables

**Table 1:** Eight different grades of typical road roughness.

Road Grade	*G_q_*(*n*_0_) × 10^−6^ (m^3^), *n*_0_ = 0.1 (m^−1^)		
	Lower Limit	Geometric Mean	Upper Limits
A	8	16	32
B	32	64	128
C	128	256	512
D	512	1024	2048
E	2048	4096	8192
F	8192	16,384	32,768
G	32,768	65,536	131,072
H	131,072	262,144	524,288

**Table 2:** The value of the vehicle model parameters.

Symbol (Unit)	Value	Symbol (Unit)	Value
*m_b_* (kg)	320	*n*_0_ (m^−1^)	0.1
*m_w_* (kg)	40	*c_s_* (N·s/m)	1000
*K_s_* (N/m)	2 × 10^4^	*f*_0_ (Hz)	0.1
*K_t_* (N/m)	2 × 10^5^	*W*	2

**Table 3:** Multi-mode switching decision rules.

Mode	Conditions of Determination	Determined Road Grade
Energy feeding + Comprehensive	$\forall G_{q}{\left(n_{0}\right)}_{i}\leq G_{q}{\left(n_{0}\right)}_{A2}\wedge v_{i}\leq v_{t}$	A
Comfort	$\forall \left(G_{q}{\left(n_{0}\right)}_{i}\in G_{q}{\left(n_{0}\right)}_{B}\wedge v_{i}\leq v_{t}\right)$ $\vee \left(G_{q}{\left(n_{0}\right)}_{i}<G_{q}{\left(n_{0}\right)}_{A2}\wedge v_{i}>v_{t}\right)$	B
Security	$\forall \left(G_{q}{\left(n_{0}\right)}_{i}\in G_{q}{\left(n_{0}\right)}_{C}\wedge v_{i}\leq v_{t}\right)$ $\vee \left(G_{q}{\left(n_{0}\right)}_{i}\in G_{q}{\left(n_{0}\right)}_{B}\wedge v_{i}>v_{t}\right)$	C
Comprehensive + Energy feeding	$\forall \left(G_{q}{\left(n_{0}\right)}_{i}>G_{q}{\left(n_{0}\right)}_{D1}\wedge v_{i}\leq v_{t}\right)$ $\vee \left(G_{q}{\left(n_{0}\right)}_{i}\in G_{q}{\left(n_{0}\right)}_{C}\wedge v_{i}>v_{t}\right)$	D

**Table 4:** Optimization of weight coefficient for multi−mode switching rules.

Mode	LQR Weight Coefficient Optimization Value Based on PSO
Energy feeding + Comprehensive Comfort	*q*_1_ = 1, *q*_2_ = 631, *q*_3_ = 2624
	*q*_1_ = 1, *q*_2_ = 232, *q*_3_ = 6856
Security	*q*_1_ = 1, *q*_2_ = 525, *q*_3_ = 47,590
Comprehensive + Energy feeding	*q*_1_ = 1, *q*_2_ = 579, *q*_3_ = 4476

**Table 5:** Test results of different speeds in each road section.

Road Section	Speed (km/h)	RMS(BA) (m/s^2^)	RMS(CA) (m/s^2^)	*G_q_*(*n*_0_) × 10^−6^ (m^3^)
1	10	0.4512	0.9919	108.84
1	30	0.5715	1.5667	110.14
1	60	1.1337	2.4482	106.98
2	18	0.4417	1.2196	66.92
2	36	0.7764	1.7760	65.94
2	72	0.9487	2.5729	65.39

**Table 6:** The value of the test bench parameters.

Symbol (Unit)	Value	Symbol (Unit)	Value
*m_b_* (kg)	1.93	*m_w_* (kg)	0.24
*c_s_*_max_ (N·s/m)	6	*U*_max_ (N)	50
*K_s_* (N/m)	1.2 × 10^2^	*K_t_* (N/m)	1.2 × 10^3^

**Table 7:** RMS comparison of BA of three groups of test data.

Time (s)	Passive (m/s^2^)	LQR (m/s^2^)	PSO_LQR (m/s^2^)
[0,3)	0.0604	0.0257	0.0246
[3,6]	0.0934	0.0387	0.0304

## Data Availability

Not applicable.
